# Exploring SARS-CoV‑2 Spike RBD Pockets as Targets for Generic Drugs: A Combined Computational, Biophysical, and Biological Approach

**DOI:** 10.1021/acsomega.5c05175

**Published:** 2025-08-25

**Authors:** Javier García-Marín, Clara Francés-Gómez, Alicia Forcada-Nadal, Anmol Adhav, Clara Marco-Marín, Vicente Rubio, Alberto Marina, José-Luis Llácer, Ron Geller, Sonsoles Martín-Santamaría

**Affiliations:** † 54446 Centro de Investigaciones Biológicas Margarita Salas (CIB), CSIC, 28040 Madrid, Spain; ‡ 588140 Institute for Integrative Systems Biology (I2SysBio), UV-CSIC, 46980 Paterna, Valencia, Spain; § 54426 Instituto de Biomedicina de Valencia (IBV), CSIC, 46010 Valencia, Spain; ∥ Group 739 at the IBV-CSIC of the Centro de Investigación Biomédica en Red en Enfermedades Raras of the Instituto de Salud Carlos III (CIBERER-ISCIII), 28029 Madrid, Spain

## Abstract

Coronavirus disease 2019 (COVID-19), caused by the severe acute respiratory syndrome coronavirus 2 (SARS-CoV-2), was a pandemic that killed over 6 million people worldwide, with devastating social and economic impacts still being felt today. Despite the recent and successful development of RNA vaccines, there remains a need for antiviral drugs to treat patients at risk for drug resistance, immunological disorders, or reduced treatment efficacy. In this regard, several computational approaches have been carried out to find small molecules targeting the SARS-CoV-2 Spike S protein, and drug repurposing strategies have been applied to find rapid and accessible candidates for clinical use. In this work, we conduct an exhaustive computational study of the receptor binding domain (RBD) of the spike S protein to identify and characterize druggable pockets and to identify generic drugs as blockers of SARS-CoV-2 entry. The combination of computational screening, biophysical studies in both the RBD (Wuhan-Hu-1 and Omicron BA.1 variants) and Spike protein (Wuhan variant), and the *in vitro* assays in SARS-CoV-2 Wuhan-Hu-1, Delta, and Omicron BA.1 has led to the identification of generic drugs with S protein binding properties and antiviral activity. Based on *in vitro* antiviral activity and mechanism of action analysis at the atomic/molecular level, fingolimod exhibited the most promising profile for a possible SARS-CoV-2 antiviral treatment.

## Graphical abstract

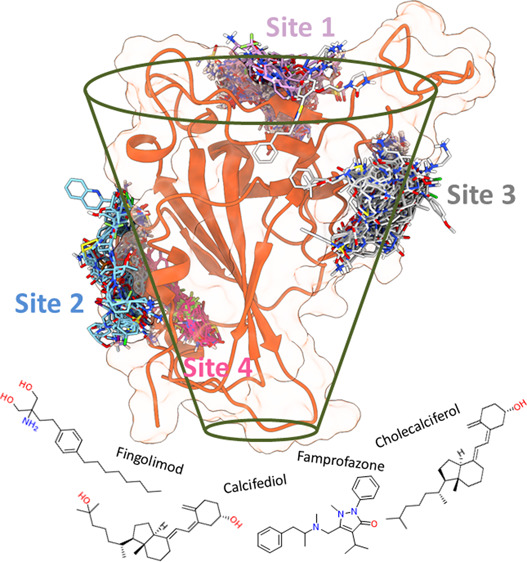

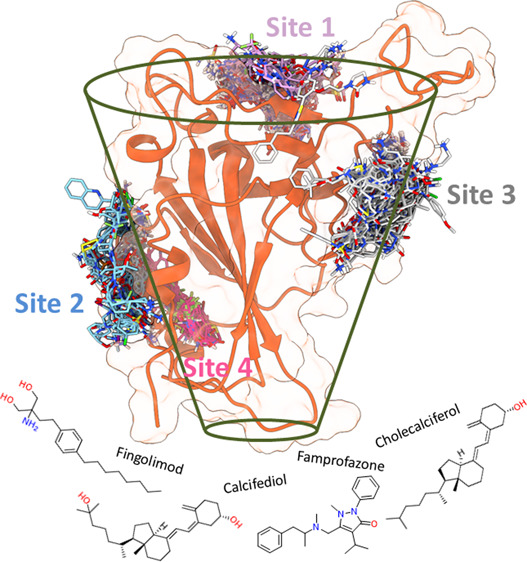

## Introduction

In December 2019, an outbreak of a new β-coronavirus spread in Wuhan (China), rapidly causing the most devastating pandemic of the XXI century thus far. The causal pathogen was named severe acute respiratory syndrome coronavirus 2 (SARS-CoV-2). SARS-CoV-2 infects human pulmonary cells, causing coronavirus infectious disease 2019 or coronavirus disease 2019 (COVID-19), a potentially fatal infection, particularly in the elderly.^1^

In response to the threat imposed by this new virus, the scientific community launched a collective effort to develop therapies in record time, which has yielded several highly effective vaccines^2^ as well as antivirals with modest efficacy for hospitalized patients.^3,4^ Especially remarkable were the worldwide efforts to generate the first vaccines against SARS-CoV-2, proving the success of the first widely used mRNA vaccines, to the point that the year 2023 Nobel Prize in Medicine or Physiology recognized the development of the technology and its value for COVID-19.^2^ Nevertheless, despite the implementation of promising COVID-19 vaccination programs, the virus has nonetheless led to millions of confirmed infections worldwide. Therefore, the discovery and development of antiviral drugs remain essential for addressing potential future coronavirus outbreaks.^5,6^ This health crisis highlighted the need to develop platforms that enable the development of vaccines and antivirals for new emerging viral diseases.

While societies have learned to live with SARS-CoV-2 as part of the seasonal endemic human viruses, the development of additional therapies that can help mitigate the cost of SARS-CoV-2 is a top priority. This is of particular relevance in the face of the rapid evolution of SARS-CoV-2, which has significantly reduced the vaccine efficacy, even eliminating the utility of some therapeutic monoclonal antibodies. In this regard, such discoveries can be facilitated by molecular probes and drug-like molecules, which provide useful tools to study biochemical processes relevant to viral replication that can be leveraged to develop new antiviral drugs.

One of the distinctive features of the efforts to better understand SARS-CoV-2 was the availability of detailed, near-atomic-resolution structural knowledge in molecular terms for the interaction of the SARS-CoV-2 spike (S) protein with the cellular receptor for the virus, the human angiotensin-converting enzyme 2 (hACE2). This advanced starting point was based on previous knowledge gained for the other two severe human β-coronavirus outbreaks, SARS-CoV-1 and MERS-CoV, and the advancements in cryo-electron microscopy (cryo-EM).^7,8^ Thus, there are a large number of publicly available high-quality structures of the S protein, its receptor binding domain (RBD), or their complex with the catalytic domain of ACE2 (the region of ACE2 that interacts with the viral RBD).^7,9,10^

Beyond their utility for understanding viral biology, high-resolution macromolecular structures enable the implementation of *in silico* drug-docking methodologies to identify novel antivirals. Indeed, *in silico* identification of potential binding sites and *in silico* testing of existing pharmaceutical libraries using molecular dynamics (MD) simulations have proven to provide a valuable avenue to identify potential drugs.^11^ Despite their high potential and flexibility for clinical development, RBD/ACE2 drug-like small-molecule blockers have rarely been reported. Capitalizing on both the availability of high-resolution structures and new MD approaches, we have computationally explored druggable pockets in the spike RBD and used them to screen a library of generic drugs to identify potential binders able to interfere with hACE2 binding and to serve as a starting point for potential therapeutics. The binding affinity of selected screened drugs was then assessed by biophysical assays, and their antiviral activity was evaluated against both the parental SARS-CoV-2 Wuhan-Hu-1 sequence and variants of concern. We have identified the drug fingolimod as a novel spike RBD binder and as a promising antiviral agent active against relevant SARS-CoV-2 variants. The novelty of our findings lies in the discovery of a drug-like small molecule with high potential to be optimized and developed as an antiviral targeting the SARS-CoV-2 spike protein.

## Results and Discussion

### Conformational Dynamics of the RBD

SARS-CoV-2 S protein can be divided into three topological domains: head, stalk, and cytoplasmatic tail.^12^ In the head, the RBD represents the key structural feature to recognize the hACE2 and trigger the viral entry into the cell. Given the importance of this protein domain, we were prompted to explore the potential druggability of the RBD region. Prior to the computational screening, we decided to study the conformational space explored by the RBD to assess the possibility of finding cryptic pockets or significant movements in the tertiary structure. We took as a starting point the geometric coordinates of the RBD bound to the human ACE2 (PDB ID: 6M0J)^9^ to run molecular dynamics (MD) simulations of the RBD in the free state in explicit water.

The root-mean-square deviation (RMSD) relative to the starting structures was calculated to examine the structural stability across the trajectories. During our simulations, we observed how the protein reached equilibrium after 100 ns with mean RMSD values that did not exceed a mean value of 1.6 Å, thus confirming the stability of the apo protein in our studies (Figure 1A). Analysis of Cα root-mean-square fluctuations (RMSF) showed a similar picture for three replicas, with average values at all positions along the RBD under 3 Å (Figure 1B,C).

**Figure 1:**
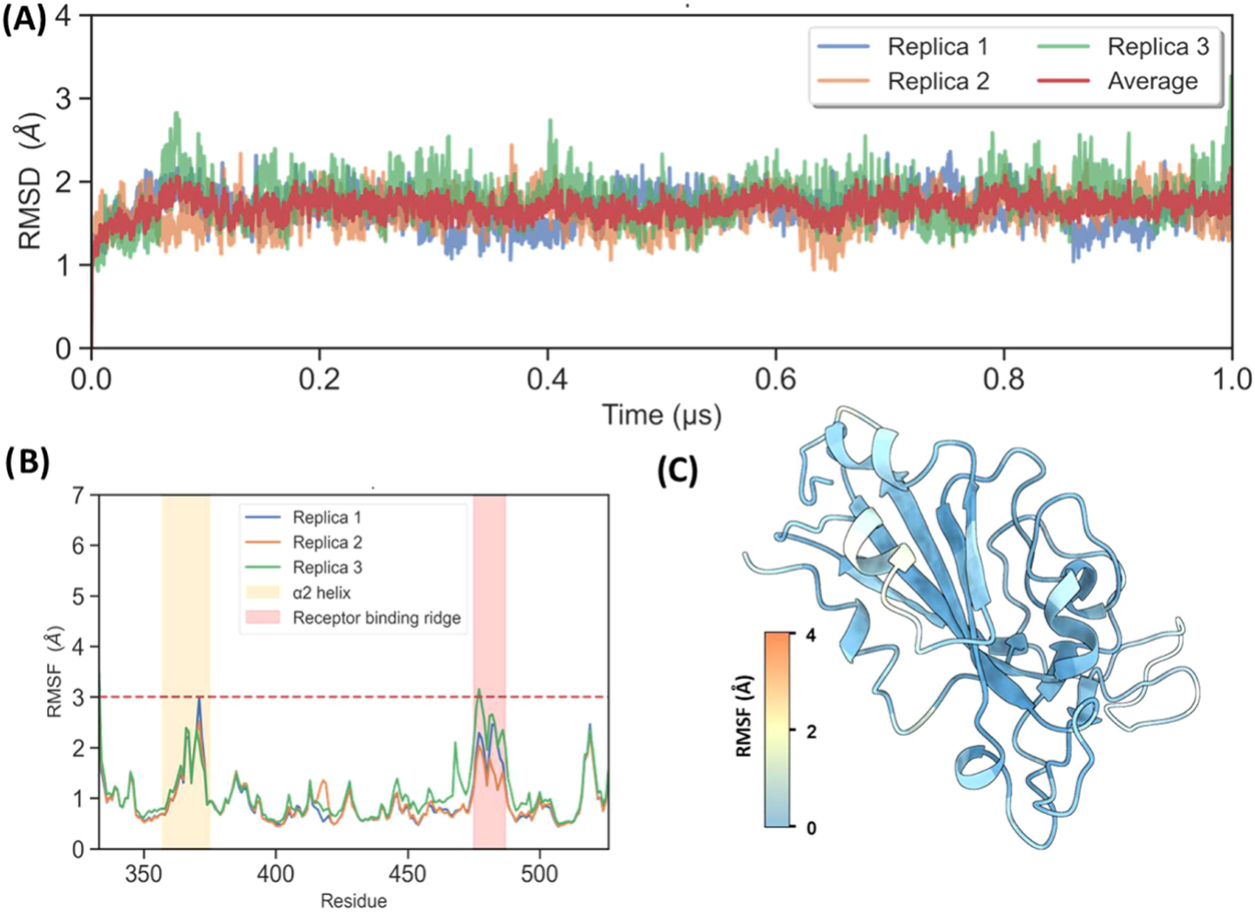
(A) RMSD plot for protein Cα atoms across MD trajectories (in red, average RMSD for three replicas). (B) RMSF plot for RBD Cα. (C) RBD cartoon representation colored according to by-residue RMSF values obtained from MD.

A detailed visual analysis of the simulation revealed that despite the stability of the main structural features of the RBD, one of the most mobile segments is the loop spanning residues 475–485, corresponding to the receptor binding ridge of the receptor binding motif (RBM). This loop is a crucial player in the process of RBD–ACE2 recognition, interacting tightly with several residues at the *N*-terminal helix of the hACE2 protease domain.^10^ Indeed, this hot spot of the RBD is an epitope for different antibodies that neutralize SARS-CoV-2 infection.^13,14^ Other RBD regions with the highest RMSF values correspond to those coils in terminal positions of our structures and the helix α2, which comprises residues Tyr365–Tyr369. A deep visual insight into the MD trajectory revealed that this helix is partially displaced along the simulation toward the α1 helix (Figure 2). This movement opens a small cavity between the RBD helixes α2 and α3. In the initial configuration, the entry to this swallowed hollow is enclosed by the “gating” Tyr369 from helix α2 that interacts with Phe377 through a t-shaped π-stacking and with Pro384 by a CH–π mediated interaction. During the MD simulation, the Tyr369 side chain rotated, breaking its initial intramolecular interactions, allowing the partial displacement of the α2 helix and the consequent pocket opening.^15^ This observation was supported by the work from Toelzer et al., in which they described that this is a free fatty acid binding pocket identified in their cryo-electron microscopy structures (PDB IDs: 6ZB5 and 6ZB4).^15^ The presence of linoleic acid in this cavity seems to relate with a high packing of the full trimeric S protein and a stabilization effect toward the closed conformation of the whole protein, thus hindering hACE2 interaction.^15^

**Figure 2:**
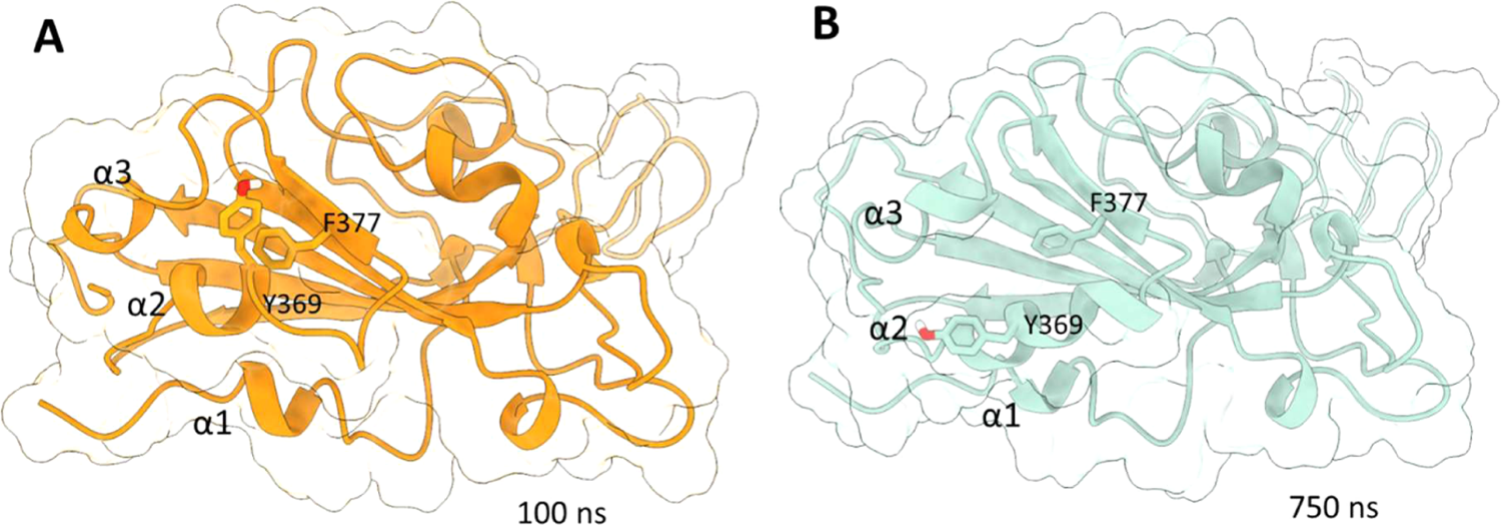
Selected snapshots at time 100 ns (A) and 750 ns (B) to illustrate the displacement of the RBD α2 helix and Tyr369 orientation.

### RBD Pocket Mapping

Several computational studies have tried to identify drug candidates that potentially bind to different regions of the S protein. *In silico* drug repurposing strategies against SARS-CoV-2 include those aimed at identifying potential ligand binders to the RBD.^16–21^ Most of these studies targeted the interface between the RBD and hACE2 in an attempt to find hypothetical small molecules able to disrupt protein–protein interactions. To the best of our knowledge, only peptides and α-helix constructs based on the hACE2 *N*-terminal helixes have been claimed as strong binders to the RBD interaction interface.^22,23^ A study based on experimental drug repurposing approaches has identified drugs with the ability to bind the RBD and to also exert antiviral activity,^24^ and only a virtual screening (VS) approach has identified natural products with RBD affinity.^21^ Our approach to find drug-like binders that can eventually decrease the SARS-CoV-2 infection by disrupting hACE2 binding relies on the rational identification of druggable pockets over the entire RBD domain, not just focusing on the RBD–ACE2 interface. For this, we decided to map the RBD crystal structure (PDB ID: 6M0J)^9^ with two different programs SiteMap^25^ and DoGSiteScorer^26^ to find druggable cavities.

In the first instance, it is important to note that none of these tools were able to identify RBM as the druggable region. This contrasts with the high number of studies and approaches to target the RBM of the spike protein (site 1 hereinafter) in an attempt to find protein–protein disruptors. However, its irregular topology and highly solvent-exposed surface may act as a counterpart for drug-like molecule binding. Indeed, druggable pockets are usually deeper, with complex shapes and high enclosures.^27^ The first putative pocket (site 2 hereafter) identified by both programs over the RBD corresponded to the region delimited by the two main helixes α1 and α2 of the RBD (Figure 3A). This cleft flanked by the two α helixes is composed of many hydrophobic amino acids including Phe338, Phe342, Phe374, Leu468, and Tyr365, which eventually could accommodate lipophilic ligands. In this pocket, there is also an Asn343 residue, which presumably is attached to a complex glycan chain *in vivo*.^28,29^ The presence of this complex sugar could exert a crucial effect in the binding process of ligands by shielding the unoccupied pocket or, in contrast, enclosing it once a ligand is bound.

**Figure 3:**
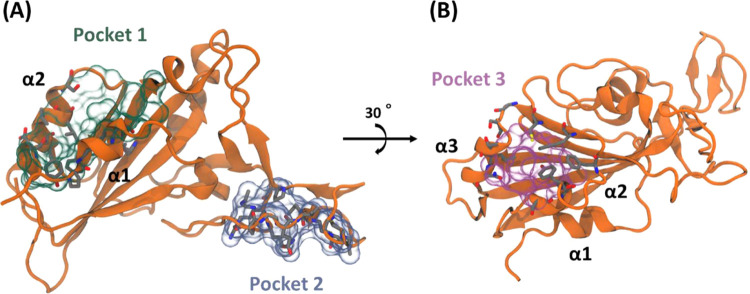
RBD view showing the three binding pockets identified by the SiteMap and DoGSiteScorer programs. For docking calculations, pockets 1, 2, and 3 are named as sites 2, 3, and 4, respectively.

A second potential drug-binding pocket (pocket 2; site 3 hereinafter) was detected by both programs, forming a narrow cavity spanning residues 458–479 (Figure 3A) close to the RBM ridge (residues 471–491). This region is formed by several coils and loops, so high mobility in this region is expected as can be deduced by the study of b-factors in the crystal structure (PDB ID: 6M0J).^9^ This fact, in conjunction with the high cavity exposure, could represent a drawback for small-molecule binding. Nonetheless, this pocket presents a higher content of polar amino acids compared with those observed in site 1, including, among them, Arg457, Lys458, and Glu471, three charged residues that could act as anchor points to establish strong intermolecular interactions.

During our MD simulation studies, we identified a possible third pocket (site 4 hereinafter, Figure 3B) that was not present at the starting 3D geometry. This pocket was reported as a linoleic-acid binding region, as aforementioned.^15^ However, during the initial mapping of the RBD, neither SiteMap nor DoGSiteScorer identified this cavity as a druggable binding pocket. This fact may be due to the 3D geometry employed for the computational analysis, which was obtained from the X-ray structure corresponding to PDB entry 6M0J (see Experimental Section). Using a snapshot from this conformation, only DoGSiteScorer was able to detect the pocket, with a good druggability index (0.8), pointing to the interest of this region as a possible drug-binding pocket. This cavity, termed site 4, was considered for subsequent virtual screening studies.

Finally, to confirm the accessibility of these sites for virtual screening, we analyzed their location within the context of the whole spike protein. To this aim, we superimposed our pockets onto the full, glycosylated structure of the S protein in complex with hACE2, obtained from the CHARMM-GUI Archive (COVID-19 Proteins Library) (Figure S1). This analysis confirmed that despite the complex glycan shield, all three pockets remain accessible to the small-molecule ligand, thus confirming their interest for virtual screening.

### Virtual Screening of Drug Libraries and MD Simulations

Considering the data obtained by our previous analysis, we decided to carry out an *in silico* or virtual screening (VS) campaign aimed at discovering potential binders to the RBD, which eventually could be used as antivirals. As drug development is a long way for a wide health emergency, drug repurposing approaches arise as excellent strategies in drug discovery. Thus, we employed our customized *in-house* library of up to 2951 compounds, which collects several approved drugs as well as vitamins, and other interesting bioactive molecules in use.^30^ VS was performed using Glide and FlexX, focusing on the three identified pockets during RBD mapping as well as in the PPI RBD/ACE2 interface (Figure 4, Tables S1 and S2). Given that docking approaches and their associated scores are inherently limited by a simple and static representation of the receptor–ligand binding phenomenon, we decided to refine the binding modes generated by extensive molecular dynamics simulations (MD). The resulting RDB–ligand complexes, obtained from virtual screening, were submitted to two 1 μs replicas of unbiased all-atom molecular dynamics simulations in order to study the stability inside the proposed binding site and to explore possible conformational changes in the RBD. The drugs finally selected for MD simulations in complex with the S protein RBD are listed in Figure S2 and Table S3.

**Figure 4:**
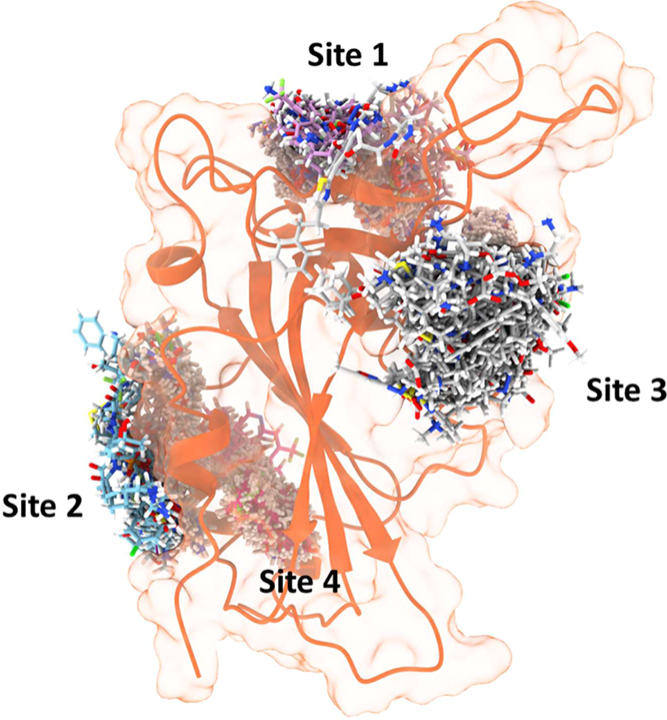
Superimposition of the top 29 hits from Glide and FlexX obtained during virtual screening campaigns over the RBD surface in each of the four studied binding sites.

#### Virtual Screening in Site 1

The RBM comprises the region that participates in the recognition and binding with hACE2. This interface is a protein–protein interaction that has been extensively explored by computational methodologies before but without providing accurate experimental support of the proposed hits.^16,17,31^ Only a few recent works report small molecules that target the RBD (and/or ACE2) and could act as protein–protein interaction disruptors.^21,32^ Considering this, targeting this region of RBD by drug-like molecules seems to be difficult. In an initial docking analysis, Glide led to 29 hit candidates with scores ranging from −5.274 to −7.733 kcal/mol (Figure 4 and Table S1) and FlexX led to 29 best solutions with estimated affinity in the micromolar range (Table S2). Among the screened drugs, no coincidences were found (Table S4).

Visual inspection of docked poses revealed that Glide tended to score better polar and charged molecules rather than FlexX. We also noticed the presence of some cofactors, nucleosides, and sugars among the proposed hits, which were discarded, as they are not interesting molecules as drug repurposing candidates. Poses tended to distribute along the RBM, without a clear pattern of interactions or a common binding mode. In light of these observations, we selected as candidates for MD simulations three compounds, fingolimod, cefamandole, and desferrioxamine B, because they presented high docking scores and targeted an extended surface over site 1. MD simulations were analyzed by means of RMSD monitoring and visual inspection (residence time, Table S3, RMSD site 1), revealing that the three molecules abandoned the binding site 1. Cefamandole is an antibiotic that has shown low antiviral effect but without reporting a RBD-mediated mechanism.^33^ This is in accordance with our simulation, where it escaped easily from site 1 (see Table S3, residence time) despite being one of the top-scoring molecules. Remarkably, in the case of fingolimod, during one of the replicas, the molecule ended up bound to site 2, once it has abandoned the initial binding site after 215 ns, until the end of the simulation (see RMSD in Figure S3). Therefore, from the screening at site 1, no small molecule was selected for *in vitro* testing.

#### Virtual Screening in Site 2

Docked scores obtained here were higher than those for site 1 for both docking procedures, suggesting a better (predicted) druggability of site 2 (Tables S1 and S2). Given that the α2 helix is an important glycosylation site,^12,34^ we discarded docked results obtained by Glide where the ligand was interacting with Asn343: FAD, cefalonium, octreotide, and ocphyl. No coincidence molecules between Glide and FlexX were found in this second search; however, a few chemotypes were identified (Table S5). Several β-lactamic antibiotics were selected by Glide (cefalonium, cefonicid, cefoxitin), while FlexX chose adrenergic drugs like salmeterol or betaxolol. Interestingly, FlexX identified fingolimod as a site 2 binder, reinforcing our previous observations arising from the MD simulation on site 1. We realized that FlexX also selected calcifediol as a putative binder and other vitamin D metabolites below the top 29 poses. This fact was particularly remarkable because several studies have reported that calcifediol and vitamin D metabolites may improve COVID-19 outcomes and patient perspectives by decreasing the acute respiratory distress syndrome.^35–37^ However, to the best of our knowledge, no specific target has been suggested for these compounds, and our results might point at the spike protein RBD as one possible target. After analysis and visual inspection of the docked results from both VS protocols, several candidates were selected for MD simulations of RBD/ligand complexes in site 2 (Table S3): banzel, nabumetone, catechin, hesperetin, calcifediol, ergocalciferol, fingolimod, betaxolol, and salmeterol. After the MD simulations, eight of the nine hit compounds selected remained inside site 2 although experiencing some fluctuations depending on the compound (see the RMSD in Figure S4). Except for salmeterol, fingolimod, and vitamin D metabolites, all compounds exhibited more than one differentiated binding mode, different from the starting docked pose, shifting alongside the hydrophobic cleft. Ligand binding is mainly led by π-stacking interactions with aromatic side chains of Phe338, Phe342, Phe374, and Trp436, as well as van der Waals interactions with hydrophobic amino acids (Figure 5). Some compounds establish occasional hydrogen bonds during simulations, especially with Asp264 backbone atoms and Ser371 side chain. Fingolimod, salmeterol, calcifediol, and ergocalciferol showed the most stable binding modes across the MD simulation according to RMSD values and visual inspection.

**Figure 5:**
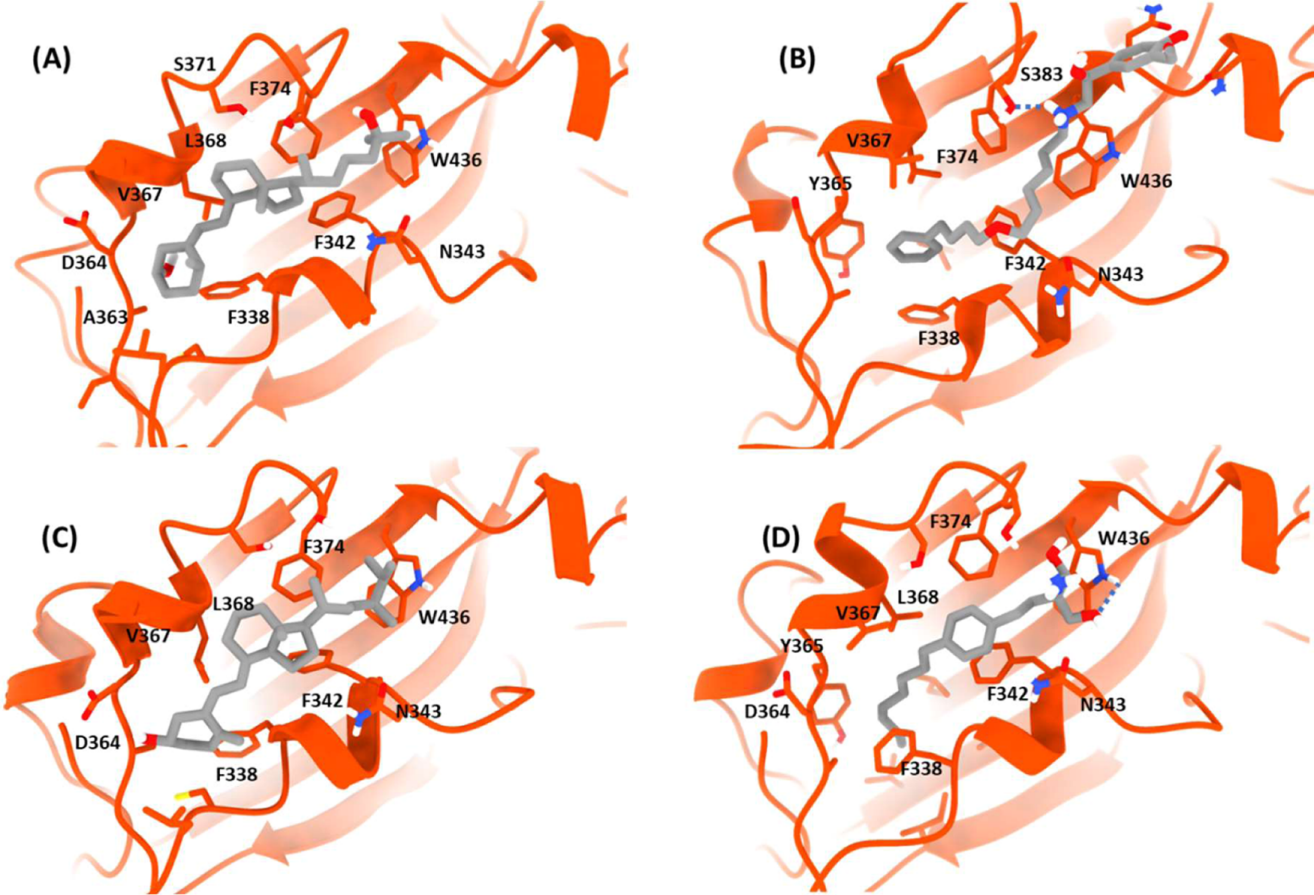
Predominant binding modes observed during 1 μs MD simulations of most stable compounds: (A) calcifediol, (B) salmeterol, (C) ergocalciferol, and (D) fingolimod. Amino acids neighboring the ligands are depicted as sticks with nonpolar hydrogens hidden to facilitate visualization.

In light of these observations for site 2, we decided to get a better insight into the role that Asn343 glycan could play in the recognition between RBD and our hits. The sugar shielding role over the SARS-CoV-2 S protein has been extensively studied by means of experimental and computational approaches so far.^34,38,39^ This glycosidic coating protects pathogens from immune response and may act as a shield for those drugs targeting this protein. Moreover, works from Amaro et al. have proved that glycans stabilize the open conformations of the RBD, and Asn343 glycan seems to be directly implicated in the pathway for the spike opening process.^12,38^ Taking all of these observations in mind, first, we visualized the MD trajectories generated, deposited in the COVID-19 Molecular Structure and Therapeutics Hub and the CHARMM-GUI Archive-COVID-19 Proteins Library of open-state S protein.^40,41^ We observed that Asn343 sugars here simulated exhibited great mobility, interacting with nearby residues, overall considerably hindering the accessibility to site 2.

We simulated the RBD attached to a complex glycan in Asn343, as previously described in other works.^12,34^ Along the simulation, this branched oligosaccharide partially occludes the entry of site two in both our replicas. However, also partially accessible conformations able to bind small molecules in this pocket were observed. Furthermore, it is important to consider that, in the context of a fully glycosylated S protein, this glycan interacts with the neighboring glycans and adjacent RBD on the trimeric protein, as has been demonstrated by studies of Amaro,^12,38^ so the real accessibility to this site may be even higher, especially in the closed-state S protein. Additionally, it is important to consider that there is a high variety and complexity in the glycosylation patterns present in the virus envelope, varying the possible outcomes and modeling approaches. In order to study the influence of the binding of a small molecule, we performed MD simulations of the glycosylated RBD (glyRBD) in complex with calcifediol, one of our screened drugs with more stable binding modes. Similar to the complex with the RBD, calcifediol remained stable inside the glyRBD site 2. However, in this case, we observed higher fluctuations in RMSD values (Figure 6), in part due to weak van der Waals interactions established with the CH groups present in the carbohydrate units attached to Asn343, and occasional hydrogen bonds that destabilize calcifediol inside the pocket. This simulation was set as a proof of concept, and its results may suggest that, despite the presence of a glycosylation site near site 2 in RBD, ligand binding may be allowed. Based on all of these findings, we decided to select for *in vitro* screenings, all molecules that have remained bound to the RBD in our simulations.

**Figure 6:**
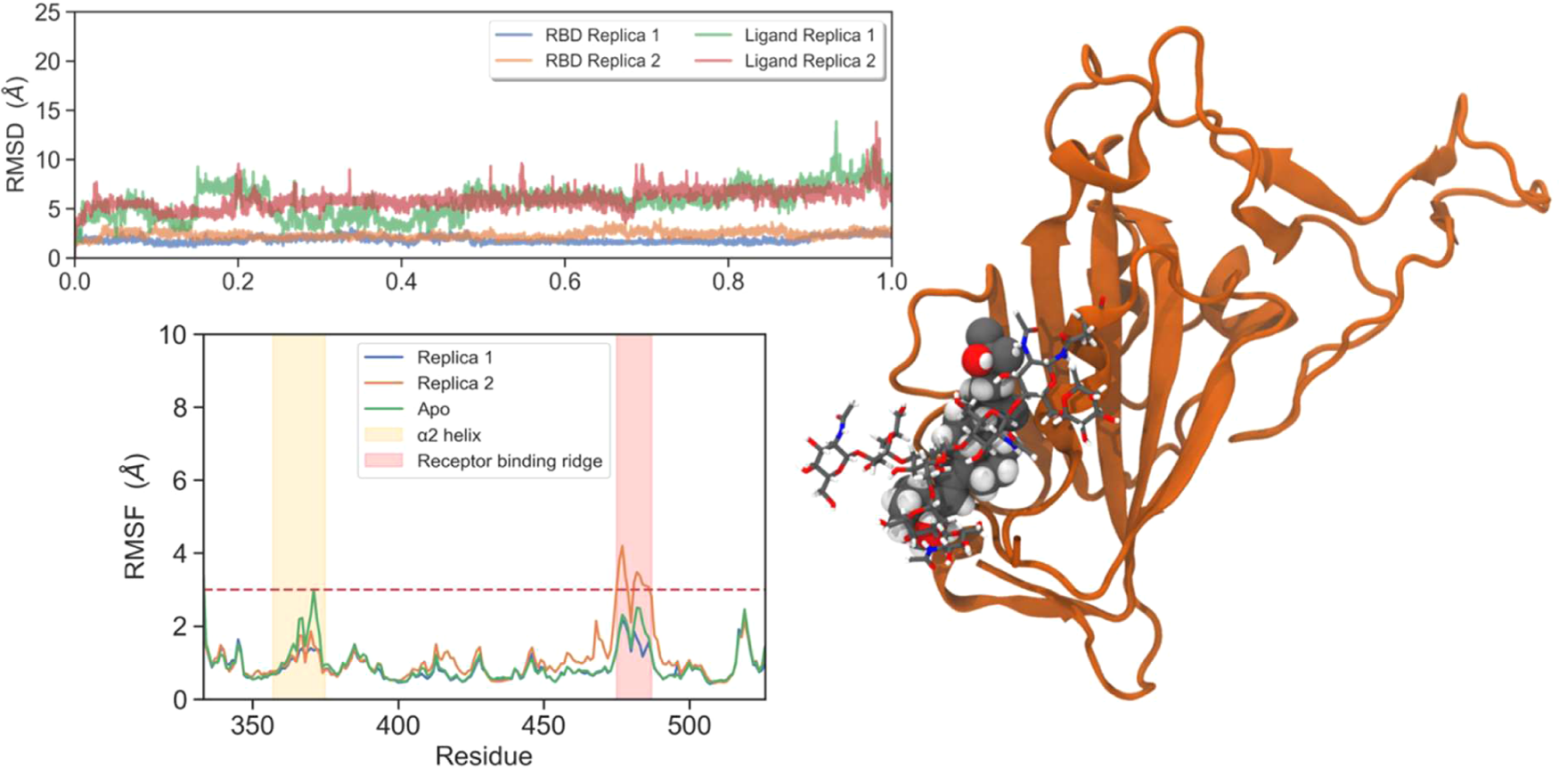
RMSD and RMSF plots of glyRBD-calcifediol simulations. Representative snapshot showing the calcifediol bound to RBD site 2 and covered by the glycan coat attached to Asn343.

#### Virtual Screening in Site 3

This cavity is allocated in a neighboring region to the receptor binding ridge, and it is highly accessible in the open state of the S protein (Figures 4 and S1). The predicted scoring values obtained by Glide and FlexX were worse compared with site 2 and had a similar tendency for site 1 (Tables S1 and S2). Among the 29 hits proposed by both docking programs (Table S6), no coincidences were found; however, more polar ligands were proposed for this site. This may reflect the fact that in site 3 there is an important content of charged amino acids such as Lys462, Glu465, Arg466, and Asp467 as well as other polar residues, given the high solvent exposure surface in this region. These results may suggest a lower druggability for this pocket in comparison to site 2. In any case, three molecules were finally selected for further studies by means of MD simulations of the corresponding RBD/ligand complexes: iohexol, methoxamine, and glafenine.

The latter unbound from site 3 easily after a few nanoseconds in both replicas; however, iohexol and methoxamine exhibited intermediate behavior. Thus, in one simulation for each candidate, compounds liberated from the binding site easily, but in the second replica both remained bound to the RBD for a long time (see RMSD in Figure S5). In this case, compounds shifted away from the binding site to the neighboring region of the receptor binding ridge, exploring more stabilizing interactions. Nevertheless, these compounds finally broke their contact with the RBD and unbound from its surface. The observations arising from the MD simulations are in good accordance with the docking-predicted binding energy for this region, suggesting poor expectations for modulation by small molecules in this site, and supported by the lack of stable binding modes inside the pocket during MD. Given that, we did not select any candidate for *in vitro* testing from the results generated herein for site 3.

#### Virtual Screening in Site 4

As aforementioned, from the analysis of our MD simulations, we observed the existence of site 4 and it was experimentally reported during our investigations.^15^ We performed docking calculations (top-ranked screened molecules in Table S7) with favorable predicted energy binding ranges (depicted in Tables S1 and S2), considerably higher than those obtained at sites 1 and 3, and only comparable to site 2 predictions. Indeed, SeeSAR estimated picomolar activity for top-ranked solutions, while Glide docking scores improved up to −3 kcal/mol compared to values obtained in the second-ranked docked solutions in site 2. This result was also supported by the presence of fatty acid ligands in this pocket, as experimentally determined by cryo-EM (PDB IDs: 6ZB5 and 6ZB4).

On the other hand, in this case, both docking tools found some shared molecules in the top-ranked molecules like famprofazone, flupentixol, and fulvestrant. Docking with FlexX, also predicted as putative binder vitamin D3 (cholecalciferol), was selected for further studies due to the interest of vitamin D in SARS-CoV-2 treatment as already stated. After careful visual inspection of docking results, we also selected oxyphenomium, sertindole, trazodone, antrafenine, and toremifene for MD simulations.

Trajectories generated showed moderately stable complexes between the RBD and proposed ligands in both replicas (see RMSD Figure S6). In addition, different binding modes were observed for most ligands, but all remained bound to the protein during the simulation time (Figure 7). Molecules are stabilized by an extensive network of van der waals and π–π interactions with residues present in this site (Tyr365, Tyr369, Phe374, Phe377, Tyr380), as well as by CH–π interactions, in a manner similar to that of eicosanoic acid (PDB ID: 6ZB5).^15^ Despite transient hydrogen bonds between polar groups and RBD backbone atoms, which could be observed in some cases, stabilization in this pocket is provided mainly by hydrophobic interactions.

**Figure 7:**
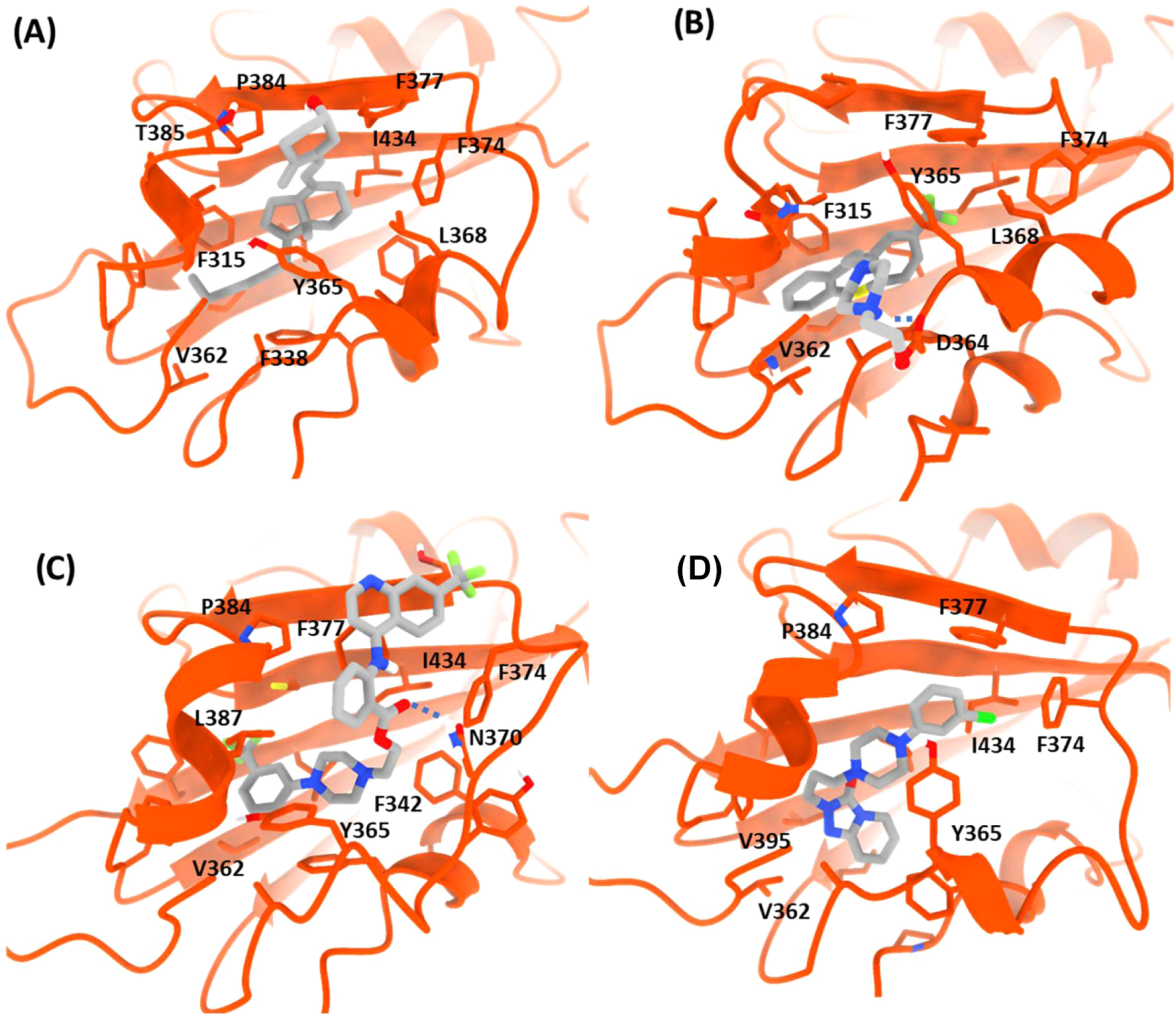
Detailed views of the (A) cholecalciferol, (B) flupentixol, (C) antrafenine, and (D) trazodone binding modes in complex with the RBD in site 4 during MD simulations.

Considering that the proposed candidates showed stable protein–ligand complexes in our MD simulations, we purchased all of the selected candidates except antrafenine (not commercially available) to carry out biophysical binding assays and *in vitro* experiments.

### Biophysical Studies of the Binding to S Protein

The compounds identified by virtual screening followed by molecular dynamics as potential binders to the RBD were tested in binding assays to the recombinantly produced RBD of the spike protein, as the key region responsible for the initial interaction with the ACE2 receptor, as well as the S protein (S:HexaPro) from both the Wuhan-Hu-1 and Omicron BA.1 variants, as indicated. The recombinant proteins were expressed using a baculovirus/insect cell system, which is crucial for proper glycosylation since both the spike protein and its RBD are glycosylated in the native viral context. The interaction tests included thermal shift assays (thermofluor) for detection of the binding, followed by microscale thermophoresis (MST) to determine not only the binding of the ligands to the RBD or S protein but also to determine if any of them hampers the interaction of these SARS-CoV-2 proteins with ACE2.

Figure 8A illustrates Thermofluor results showing the SYPRO Orange fluorescence profiles of the RBD_Wuhan_ in the absence of any ligands, along with observed curve shift toward lower temperatures revealing binders among the ten tested compounds, added at 0.5 mM concentration. In these assays, protein unfolding is monitored by tracking the fluorescence increase as the temperature of the solution is gradually increased at a constant rate. Ligand binding can influence the thermal stability of the protein, which is detected through changes in the fluorescence profile, quantified by shifts in the melting temperature (*T*_m_), the temperature at which fluorescence reaches 50% of its maximum change. The shift in *T*_m_, either in the sense of stabilization or destabilization, is considered an indicator of the binding of the compound to the molecule. It is important to note that only compounds dissolved in DMSO were included in this analysis. Among these, flupentixol, fulvestrant, and sertindole exhibited intrinsic fluorescence in the absence of protein, making it impossible to analyze them using this method. Figure 8B presents a similar analysis for the S protein, while Figure 8C,D shows for the Wuhan-Hu-1 variant protein targets the differences in *T*_m_ values compared to the ligand-free protein. Significant shifts in thermal stability were observed for two compounds—fingolimod (compound 7) and toremifene (compound 15)—affecting both the RBD and S proteins of the Wuhan-Hu-1 variant. For the other compounds (except for iofendylate in the Spike protein, compound 5), there was little to no change in the thermal stability of the RBD and S proteins, indicating that most tested compounds did not significantly bind. However, the data suggests a potential binding interaction for toremifene and, more notably, fingolimod with both the RBD and S proteins.

**Figure 8:**
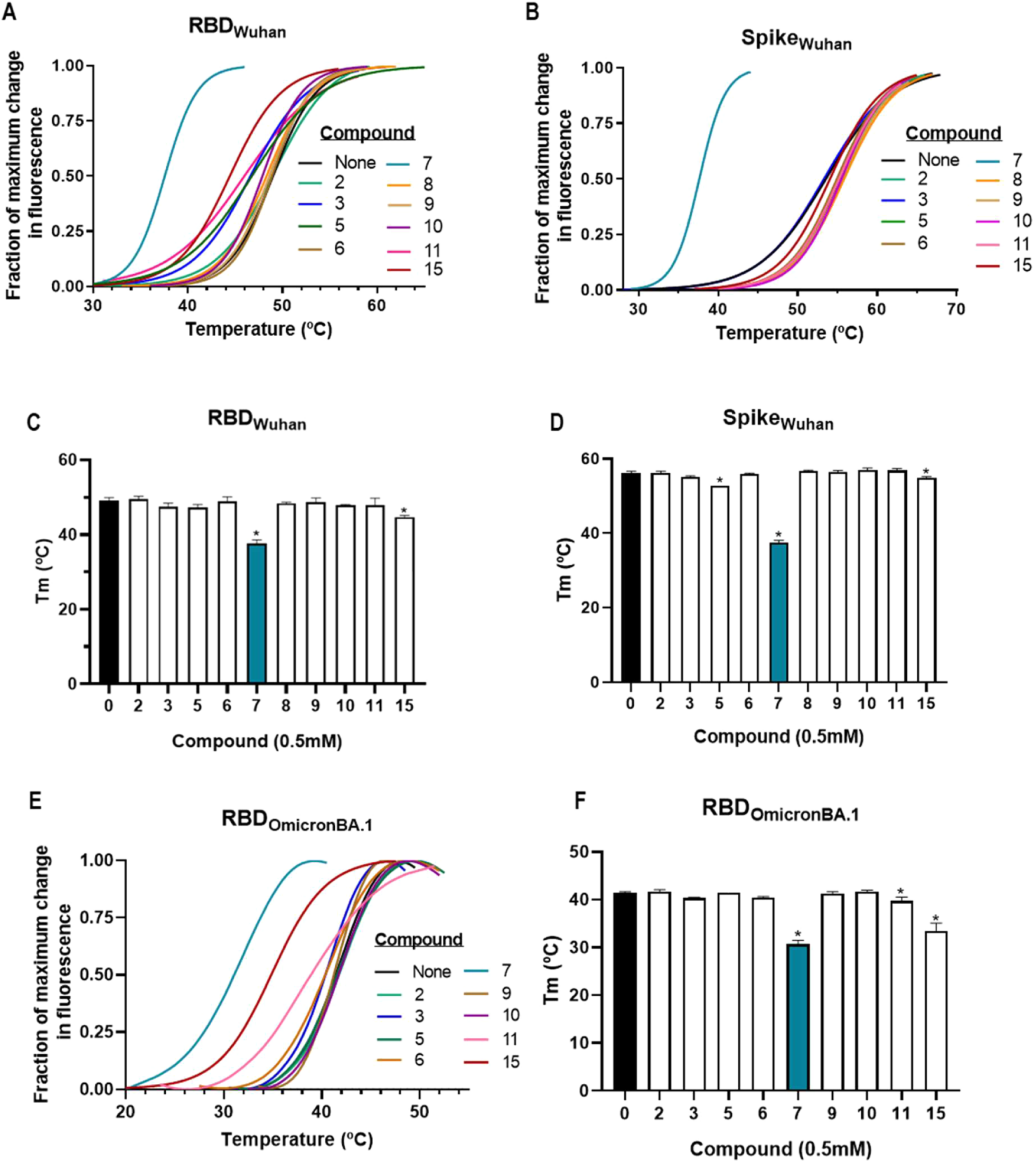
Impact of the tested compounds on thermofluor profiles and *T*_m_ values of RBD (Wuhan-Hu-1 and Omicron BA.1 variants) and Spike (Wuhan-Hu-1 variant) recombinant proteins. Panels (A–E) display the sigmoidal fitting of the fluorescence profiles (mean of at least two experiments, each one with three replicates) with gradual increase in temperature for RBD (A) and Spike protein (B) of the Wuhan-Hu-1 variant, or RBD of the Omicron BA.1 variant (E), each one in the presence of 500 μM of the indicated compounds (“None”, no compound). Fluorescence changes are given as a fraction of the maximum observed transition. Panels (C–F) show the temperatures corresponding to 50% of the maximum fluorescence change (*T*_m_; means ± SD for ≥2 determinations) for RBD (C) or Spike protein (D) from the Wuhan-Hu-1 variant and for RBD from the Omicron BA.1 variant (F) in the absence (“0”) or presence of 0.5 mM of the indicated compound. Statistical significance (Dunnett’s multiple comparison test versus the “0” column in one-way ANOVA) is indicated by * (*P* ≤ 0.0001). Compound numbering corresponds to 2: bisoprolol, 3: hesperetine, 5: iofendylate, 6: calcifediol, 7: fingolimod, 8: salmeterol, 9: nabumetone, 10: betaxolol, 11: catechin, and 15: toremifene.

We also conducted thermofluor assays using the RBD of the Omicron BA.1 variant in the presence of the selected compounds to assess whether binding behaviors differed when applied to the Omicron BA.1 variant (Figure 8E). The assays revealed a decrease in the thermal stability of the Omicron BA.1 RBD compared to the Wuhan-Hu-1 RBD. Despite this, the results for both variants upon compound addition were quite similar. As with the Wuhan-Hu-1 RBD, fingolimod and, to a lesser extent, toremifene caused significant shifts in the thermal stability of the Omicron BA.1 RBD (Figure 8F).

Since fingolimod induced the largest shift in *T*_m_ for both the RBD and Spike proteins of the Wuhan-Hu-1 and Omicron BA.1 variants, we further examined the concentration-dependent effects of fingolimod on the RBD of both variants as well as on the Spike protein of the Wuhan-Hu-1 variant. The *T*_m_ shifts induced by fingolimod were consistent across all concentrations tested for both variants (Figure 9).

**Figure 9:**
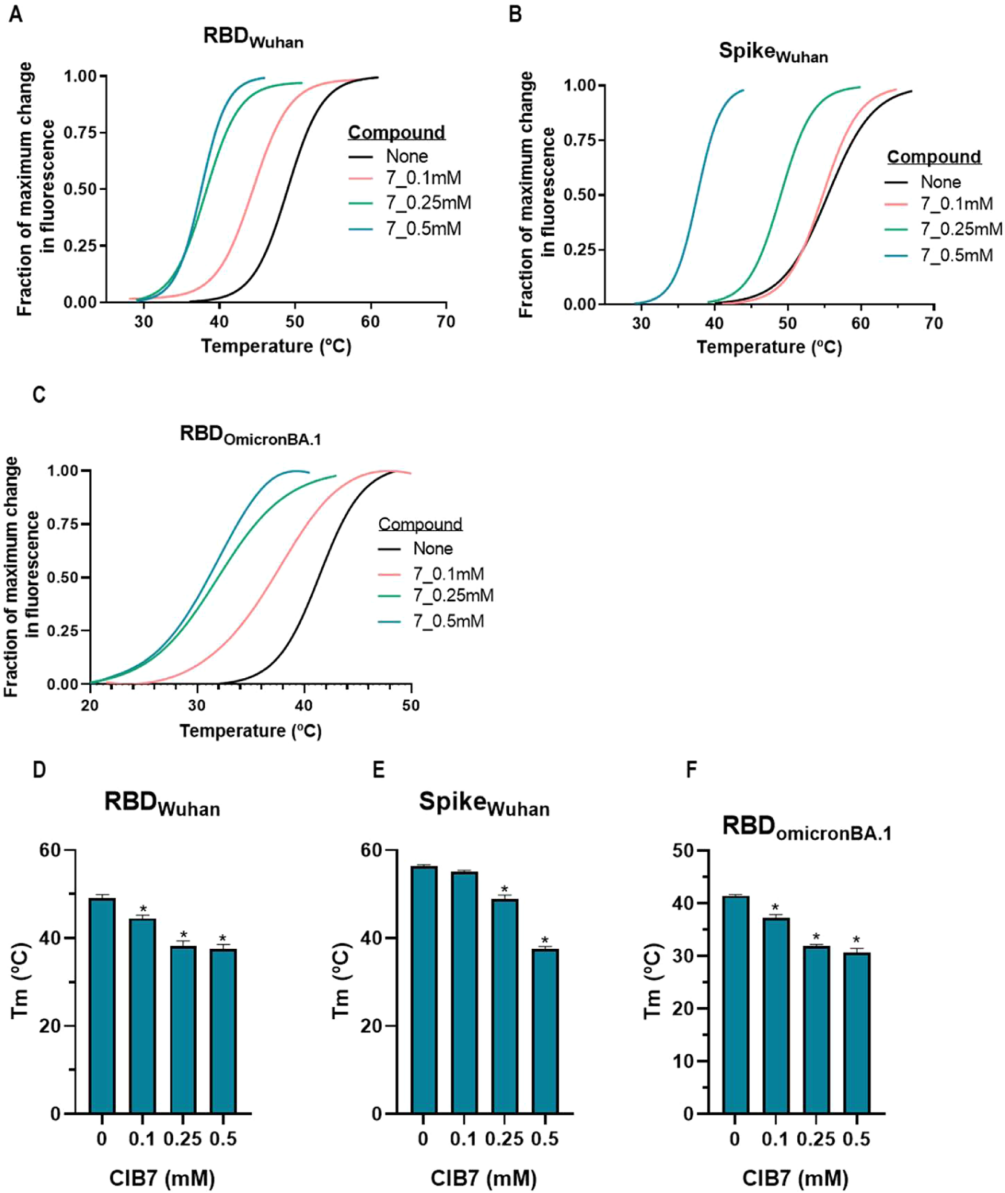
Impact of different concentrations (as indicated) of compound 7 (CIB7; fingolimod) on (A–C) thermofluor profiles and (D–F) *T*_m_ values of (A, D) RBD Wuhan-Hu-1 variant, (B, E) Spike Wuhan-Hu-1 variant, and (C, F) RBD Omicron BA.1 variant. Except for the variable concentrations of fingolimod, all other details are given in the legend of Figure 8. *T*_m_ values are given as means ± SD (≥2 determinations). Statistical significance (Dunnett’s multiple comparison test versus the noncolumn in one-way ANOVA) is indicated by * (*P* ≤ 0.0001).

These findings indicate that two compounds, fingolimod and, to a lesser degree, toremifene, interact with the RBD of both the Wuhan-Hu-1 and Omicron BA.1 variants and, as expected, with the complete S protein (tested with the Wuhan-Hu variant), triggering significant shifts in thermal stability. These shifts in thermal stability do not damage the architecture of the proteins, as judged by negative-stain electron microscopy of spikes incubated in the presence of 500 μM fingolimod (Figure S7).

To corroborate these thermofluor results, we performed additional compound-binding assays using microscale thermophoresis (MST). MST showed that only fingolimod, toremifene, famprofazone, cholecalciferol, and oxyphenomium (the last three not tested in Thermofluor assays) showed binding affinities to RBD_Wuhan_ below 1 mM (*K*_D_ values 36, 51, 115, 315, and 416 μM, respectively; Table 1). For the rest of the compounds, either we did not detect binding or the adjustment indicated *K*_D_ values higher than 1 mM that could not be determined because of limitations such as solubility of the compound and highest concentration of the solvent, which ideally should be less than 5%. MST with protein S_Wuhan_ confirmed that the lowest *K*_D_ values were obtained with fingolimod, toremifene, cholecalciferol, famprofazione, and oxyphenomium (*K*_D_ values 18, 26, 64, 88, and 92 μM, respectively; Table 1), although with this protein, we could also determine *K*_D_ values for another 5 compounds within the range of 481–927 μM (Table 1).

**Table 1:** Affinities of Wuhan-Hu-1 and Omicron BA.1 Variants of the RBD and S Proteins for Different Tested Compounds Determined by Microscale Thermophoresis

			*K*_D_ values (μM)^a^ ^,^ ^b^ ^,^ ^c^			
			Wuhan-Hu variant		Omicron variant	
compound	code	solvent	RBD_Wuhan_	S_Wuhan_	RBD_BA.1_	S_BA.1_
ergocalciferol	CIB 01	EtOH	NB	>1000	NB	NB
bisoprolol	CIB 02	DMSO	NB	>1000	>1000	NB
hesperetine	CIB 03	DMSO	>1000	>1000	NB	NB
trazodone	CIB 04	MeOH	NB	NB	NB	NB
iofendylate	CIB 05	DMSO	NB	>1000	NB	NB
calcifediol	CIB 06	DMSO	>1000	716 ± 82	>1000	NB
fingolimod	CIB 07	DMSO	36 ± 3	18 ± 4	193 ± 46	56 ± 12
salmeterol	CIB 08	DMSO	>1000	481 ± 37	>1000	561 ± 129
nabumetone	CIB 09	DMSO	NB	NB	NB	NB
betaxolol	CIB 10	DMSO	NB	NB	NB	NB
catechin	CIB 11	DMSO	>1000	927 ± 76	NB	>1000
cholecalciferol	CIB 12	EtOH	315 ± 23	64 ± 8	612 ± 101	155 ± 33
flupentixol	CIB 13	DMSO	NB	656 ± 62	NB	>1000
fulvestrant	CIB 14	DMSO	NB	738 ± 56	NB	NB
toremifene	CIB 15	DMSO	51 ± 5	26 ± 5	156 ± 47	72 ± 21
sertindole	CIB 17	DMSO	NB	>1000	NB	NB
oxyphenomium	CIB 19	Water	416 ± 45	92 ± 9	571 ± 98	163 ± 31
famprofazone	CIB 20	EtOH	115 ± 17	88 ± 9	369 ± 91	146 ± 42

a Results given as mean ± SD.

b NB: No binding detected subject to low signal-to-noise ratio of the thermophoresis run.

c >1000: Due to limitations in maximum concentration for titration and solvent.

Likewise, the compounds were also subjected to the same tests with MST for the Omicron BA.1 variant proteins. We observed that the affinity for all of the compounds was lower than for the corresponding Wuhan-Hu-1 variant proteins (Table 1). However, the best binding compounds to both the RBD and spike remained fingolimod and toremifene (*K*_D_ values ranging from 56 to 193 μM). The other compounds that bound to the RBD of the Wuhan-Hu-1 variant also bound to the RBD and S proteins of the Omicron BA.1 variant, although with reduced affinity (Table 1), whereas those showing no binding to the RBD Wuhan-Hu-1 variant also did not bind the RBD or spike of the Omicron BA.1 variant, except salmeterol (compound 8, Table 1), which was found to bind with low but measurable affinity to both the S_Wuhan_ and S_Omicron_ proteins, while the corresponding *K*_D_ values for the isolated RBDs exceeded 1000 μM and thus would not be measured (Table 1).

MST assays were also employed for testing whether the compounds that were bound to the target proteins interfered with the binding of these proteins to the recombinantly expressed ACE2 receptor protein (the monomeric soluble catalytic domain of ACE2). Table 2 gives results for these assays for all of the compounds tested. Many of the compounds exhibited some degree of interference with ACE2-RBD or ACE2-Spike complex formation (Table 2).

**Table 2:** Interference of Some Compounds with the Interaction between the Targeted Proteins and the Human Receptor ACE2

		*K*_D_ values for binding to ACE2 (nM)^a^			
		Wuhan-Hu variant		Omicron variant	
compound	code	RBD_Wuhan_	S_Wuhan_	RBD_BA.1_	S_BA.1_
none	0	73 ± 3	15 ± 1	15 ± 2	5 ± 0.5
ergocalciferol	CIB 01	89 ± 1	50 ± 5	18 ± 2	16 ± 1
bisoprolol	CIB 02	63 ± 1	22 ± 2	22 ± 3	56 ± 8
hesperetine	CIB 03	104 ± 3	41 ± 4	11 ± 2	21 ± 2
trazodone	CIB 04	88 ± 2	36 ± 1	58 ± 5	8 ± 1
iofendylate	CIB 05	116 ± 4	78 ± 6	67 ± 8	15 ± 2
calcifediol	CIB 06	151 ± 5	188 ± 5	92 ± 10	83 ± 4
fingolimod	CIB 07	26 ± 1	116 ± 4	84 ± 8	168 ± 6
salmeterol	CIB 08	63 ± 2	156 ± 20	117 ± 11	69 ± 8
nabumetone	CIB 09	69 ± 2	11 ± 1	25 ± 3	17 ± 2
betaxolol	CIB 10	93 ± 2	18 ± 1	15 ± 2	23 ± 2
batechin	CIB 11	182 ± 3	45 ± 2	102 ± 5	57 ± 6
bholecalciferol	CIB 12	461 ± 13	73 ± 2	147 ± 8	89 ± 8
flupentixol	CIB 13	116 ± 3	8 ± 0.5	42 ± 4	62 ± 6
fulvestrant	CIB 14	133 ± 5	56 ± 1	19 ± 2	17 ± 1
toremifene	CIB 15	276 ± 12	298 ± 14	47 ± 5	211 ± 13
sertindole	CIB 17	114 ± 3	60 ± 3	28 ± 2	7 ± 1
oxyphenomium	CIB 19	515 ± 5	189 ± 13	136 ± 8	121 ± 9
famprofazone	CIB 20	172 ± 5	120 ± 3	129 ± 6	96 ± 8

a Results given as mean ± SE.

Among the four proteins tested here in these assays, S_Omicron_ was the one exhibiting the highest affinity for ACE2 (Table 2). Perhaps because of this, results with this protein proved to be best to monitor binding interference of the compounds with the ACE2 receptor. Figure S8A strongly suggests for S_Omicron_ that such interference grossly parallels the affinity of the compound for S_Omicron_. Indeed, the shape of the curve fitting to these results corresponded to the one expected for competitive inhibition of ACE2-binding by the compounds that were bound to the S protein (Figure S8). As we also found that there was good correlation between the *K*_D_ values for binding of the compounds to the S_Wuhan_ and S_Omicron_ proteins (Figure S8B), we hypothesized that more global evidence of the relatedness between the affinity of the compounds for the four ACE2-binding proteins tested here and the ability to interfere with ACE2 binding to the same proteins could be obtained if all of the results on interference of each compound with the binding to ACE2 of the four proteins could be pooled together. We did that by determining for each protein the quotient of *K*_D_^ACE2^ in the presence of 0.5 mM of each compound versus *K*_D_^ACE2^ in the absence of the compound (Table 2). Then, we determined for each compound the means of these quotients for the four proteins. Figure S8C represents the results of this calculation, showing larger relative increases in *K*_D_ for ACE2 by compounds that globally bind more strongly to the four ACE2-binding proteins (see legend to Figure S8 for further details). It is interesting that these compounds were shown computationally to bind to either site 2 or site 4, regions that are spatially distant from the ACE2-binding interface and thus unlikely to directly compete with ACE2, suggesting indirect competition via induction of conformational changes on the S protein.

### SARS-CoV-2 Variants: Molecular Modeling Discussion

Considering the results obtained by MST discussed above, we were prompted to model the interaction between the best ligand, fingolimod, and the RBD from the Omicron variant. Since the beginning of the pandemic, the ability of the virus to mutate has resulted in the emergence and spread of a number of genomic variants.^42^ With more than one million SARS-CoV-2 sequences identified up to date, Delta and Omicron were two variants of interest due to their impact on transmissibility and infectivity according to the WHO.^43^ Point mutations are distributed alongside the whole spike protein but in the case of Delta variant, two different mutations are located in the RBD, while up to 15 different substitutions are found in Omicron RBD.^44^ For the Delta virus, two characterized mutations L452R and T478K are neighboring the RBM without being implicated in the four proposed sites mentioned above. A different scenario was found for the Omicron variant, with ten mutations affecting RBM or site 1. Despite the fact that we do not propose any molecule to target this region, this mutational susceptibility along the protein–protein interaction surface could have difficulty binding potential broad-spectrum drugs against SARS-CoV-2 variants. We have also noticed some mutations affecting site 2: G339D, S371L, S373P, and S375F. Despite the remarkable number of residues affected for this small pocket, three of these substitutions lead to an increment in the hydrophobic nature of this cavity according to the Eisenberg scale^45^ and thus could hinder the union of polar or charged ligands. Since the binding mode and interaction of our selected hits for this site are governed mainly by hydrophobic interactions, no significant impact is expected in the behavior between the original Wuhan and Omicron variant. To support this statement, we selected fingolimod, which elicited the best binding affinity for this site, to undertake MD simulations of the RBD-ligand complexes (see Figure S9). As expected, the molecule remained bound to site 2 during both simulation replicas, maintaining the aliphatic tail immersed in the pocket, while the polar head fluctuated by occasional ionic interaction between the positively charged ammonium group and the carboxylate from Asp339. Overall, these simulations suggest that fingolimod can bind to both the ancestral Wuhan-Hu-1 and Omicron variants with similar affinity in site 2, in good agreement with the experimental results discussed above. Nevertheless, a systematic binding free energy analysis across distinct sites and variants would be required to rigorously assess and further elucidate these observations.

### Biological Studies of the Antiviral Activity of Selected Drugs Targeting S Protein

To validate the ability of the compounds to block SARS-CoV-2 entry, a pseudotype virus assay was employed. Briefly, vesicular stomatitis virus encoding GFP in place of its native entry protein G was produced in cells expressing the SARS-CoV-2 Wuhan S protein (VSVΔG-S_Wh_). During budding, the virus is coated with the S protein, which can mediate entry into subsequent cells in a manner that faithfully recapitulates the native virus. Various concentrations of calcifediol, cholecalciferol, camprofazone, fingolimod, and oxyphenonium were evaluated for both reduction of viral entry and cellular toxicity in VeroE6 cells (Figure 10). Cholecalciferol, famprofazone, and fingolimod showed antiviral activity in the absence of toxicity (Figure 10). On the other hand, antiviral activity could not be separated from toxicity (calcifediol) or could not be observed (oxyphenonium). The 50% concentration that reduces viral infection or cell viability can be found in Table 3.

**Figure 10:**
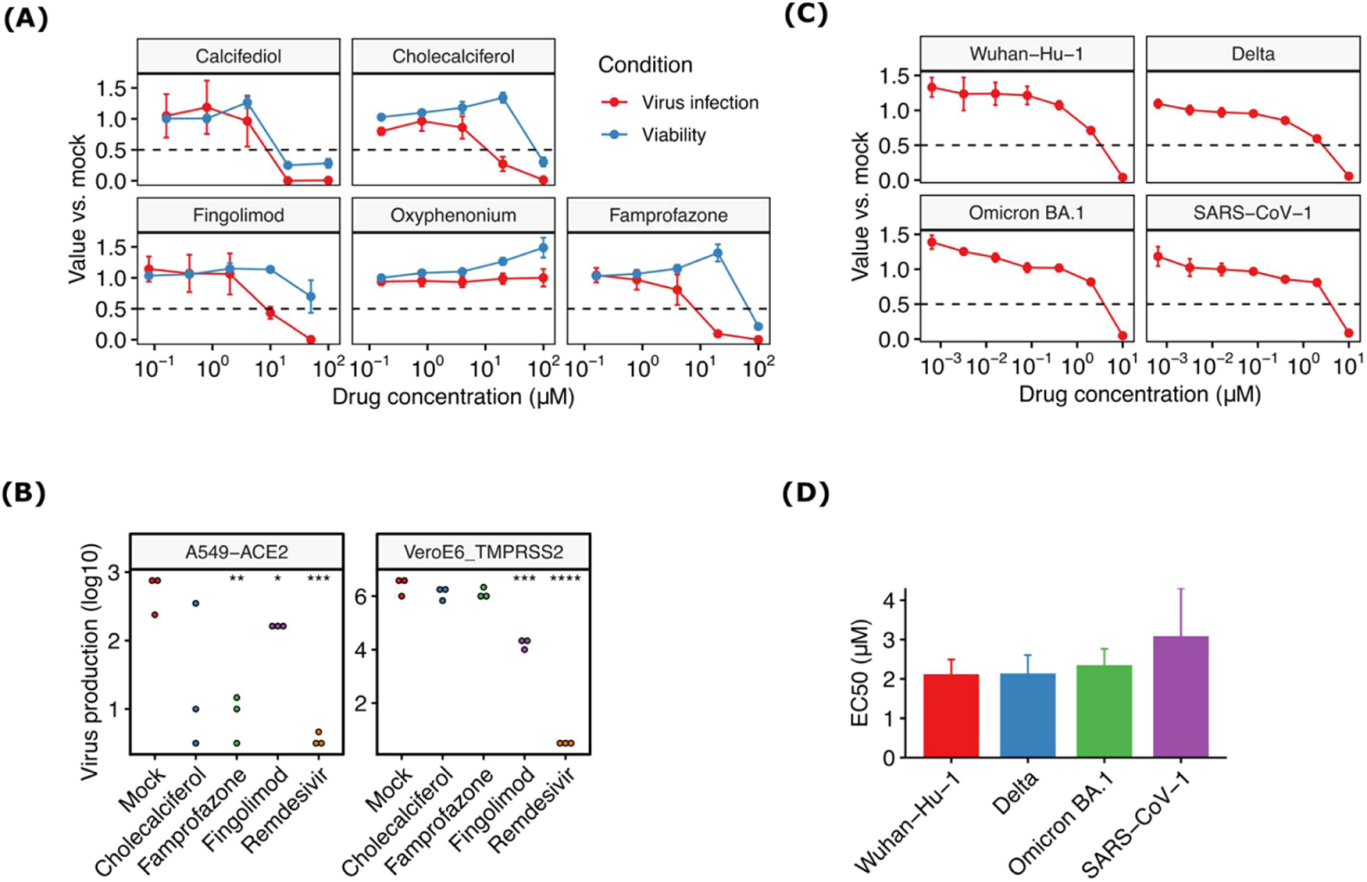
*In vitro* antiviral activity of selected compounds. (A) Antiviral activity against VSV pseudotyped with the S protein of Wuhan-Hu-1 and cellular toxicity for the indicated compounds in VeroE6 cells (*n* = 3). (B) Virus production of SARS-CoV-2 infection by the indicated compounds at 20 μM or remdesivir (30 μM) in either A549-ACE2 or VeroE6-TMPRSS2 cells. (C) Antiviral activity of fingolimod against VSV pseudotyped with the indicated S protein (*n* = 3). (D) Concentration reducing 50% of viral infection of VSV pseudotyped with the indicated S protein. All data represent the mean and standard error of 3 independent replicates. * *p* < 0.05, ** *p* < 0.01, *** *p* < 0.001, and **** *p* < 0.0001 by two-tailed *t* test on log-transformed data.

**Table 3:** Summary of Antiviral Inhibition, Toxicity, and Therapeutic Window

compound	EC_50_ ^a^ ^,^ ^b^ (μM)	CC_50_ ^c^ (μM)	therapeutic window^d^
calcifediol	6.13 ± 3.52	16.83 ± 0.5	3.34 ± 1.59
cholecalciferol	14.85 ± 4.11	83.69 ± 6.12	5.88 ± 1.37
famprofazone	7.64 ± 4.55	78.98 ± 2.75	12.95 ± 6.88
fingolimod	7.12 ± 2.25	20.36 ± 19.58	2.64 ± 2.69

a Concentration reducing infection by VSV pseudotyped with the SARS-CoV-2 Wuhan-Hu-1 S by 50% (EC_50_).

b Data reflect the mean ± SD of 3 independent experiments.

c Concentration reducing VeroE6 viability by 50% (CC_50_).

d Therapeutic window calculation: CC_50_/EC_50_.

To validate the ability of the compounds to block genuine SARS-CoV-2 and work in multiple cell lines, we infected human lung A549 cells expressing hACE2 or VeroE6 expressing the entry cofactor TMPRSS2 (VeroE6-TMPRSS2) with SARS-CoV-2 and evaluated virus production after 24 h. A single concentration (20 μM) of each compound was utilized and remdesivir was included as a positive control. As expected, remdesivir reduced viral replication significantly across both cell lines (*p* < 0.001 by *t* test on log-transformed data; Figure 10B). In A549-ACE2 cells, both famprofazone and fingolimod resulted in a statistically significant reduction of virus production (*p* < 0.05 and *p* < 0.01 by *t* test on log-transformed data, respectively), while in VeroE6-TMPRSS2, significant reduction was observed only for fingolimod (*p* < 0.001 by *t* test on log-transformed data).

Finally, as fingolimod showed promising antiviral activity in two different cell lines, we assessed the ability of the drug to inhibit infection by VSV pseudotyped with SARS-CoV-2 Wuhan, Delta, and Omicron BA.1 Spike, as well as that of the more distantly related SARS-CoV-1. A dose-dependent reduction was observed in all cases with similar 50% inhibitory concentrations (Figure 10C,D), supporting the general ability of fingolimod to block viral infection of this family. This result seems particularly of interest since fingolimod is a first-line drug to treat patients with multiple sclerosis, an immunological disorder, who were prone to suffer worse outcomes during COVID-19. Indeed, a clinical trial has been reported that provides preliminary evidence that fingolimod may be helpful in reducing readmission rates in moderate to severe COVID-19 patients.^46^

## Conclusions

The SARS-CoV-2 outbreak has highlighted the urgent need for a broad range of therapeutics to combat this and future pandemics. In this context, *in silico* approaches, including drug repurposing strategies for generic drugs, emerge as a highly advantageous strategy to reevaluate existing marketed therapeutics, including antivirals. Despite the vast number of *in silico* studies targeting the Spike protein and focusing on the receptor binding domain (RBD), the intricate topology of this motif represents a challenging target for drug-binding. We have investigated four distinct binding sites on the RBD using docking and all-atom molecular dynamics simulations, and our findings indicate that only sites 2 and 4 are suitable for drug-binding. Moreover, we combined our computational results with biophysical studies and proved that some of the candidates present moderate affinity against the recombinant RBD protein. Cellular assays demonstrated the inhibitory activity on virus replication, with fingolimod showing the most promising results, with micromolar antiviral activity similar to famprofazone and vitamin D analogues cholecalciferol and calcifediol. Our research contributes to establishing the mechanism of action for fingolimod as an anti-SARS-CoV-2 agent and to progress in the knowledge of the Spike protein modulation for the finding of new drug-like molecules to control COVID-19 disease.

## Experimental Section

### Molecular Modeling

#### SARS-CoV-2 RBD Protein Preparation

In this work, we used two different structures of the RBD to perform molecular modeling studies. First, the coordinates of the X-ray crystal structure of RBD from SARS-CoV-2 spike protein bound to human ACE2 were retrieved from PDB ID: 6M0J (chain E).^9^ For our initial purposes, *N*-acetylglucosamine bound to Asn343 was removed from the 6M0J 3D structure. As the starting structure for virtual screening on site 4, we chose RBD bound to linoleic-acid structure (PDB: 6ZB5).^15^ In this case, we retrieved the whole structure of SARS-CoV-2 Spike protein, and the RBD spanning from residues Thr333 to Gly526 was extracted from Chain A using Chimera. The resulting RBDs were then aligned in Chimera to the RBD 6M0J structure, with an overall root-mean-square deviation of 0.809 Å for Cα atoms. Both RBD proteins were prepared using the Protein Preparation Wizard included in Maestro (Schrödinger Release 2020-2: Maestro, Schrödinger, LLC, New York, NY (2020)). All ligands and solvent molecules were removed, hydrogen atoms and bond orders were assigned as well as protonation states at pH 7.4. The *N*- and *C*-termini of RBD were acetylated and amidated respectively to avoid artificially charged termini.

To prepare the glycan-bound structure of RBD, the Glycan Reader & Modeler tool was used to generate initial 3D geometries.^47^ The prepared RBD structure from the starting coordinates of 6M0J was uploaded to CHARM-GUI^48^ then, a glycan chain according to the glycan patterns previously described and modeled in the literature was attached to Asn343 (Figure S10)^12,49^

sequence:bDGlcNAc(1→2)aDMan(1→6)[bDGlcNAc(1→2)aDMan(1→3)]bDMan(1→4)bDGlcNAc(1→4)[aLFuc(1→6)]bDGlcNAc(1→)→Asn343

#### Pocket Analysis

To map the RBD surface, two tools were selected: SiteMap^25^ and DoGSiteScorer.^26^ The latter is a grid-based method that combines a vector machine model, while SiteMap uses an algorithm to determine the likeliness of a site point to contribute to tight protein–ligand interactions.^50^ The prepared RBD (PDB ID: 6M0J) was uploaded to the Protein plus web server for pocket detection with DoGSiteScorer (https://proteins.plus/). Site 4 was characterized in the same way using a snapshot taken from molecular dynamics production after 120 ns. We considered only those pockets with an appropriate size (more than 10 amino acids)^51^ identified by both softwares and with the highest scores (simple score >0.1 and Dscore >0.5, Table 4).

**Table 4:** Results from the Pocket Analysis by DogSiteScorer and SiteMap

pocket	simple score/drug score (DoGSiteScorer)	SiteScore/Dscore (SiteMap)	residues
1			receptor binding motive (437–508) 405–417, 445–457, 489–508
2	0.31/0.79	0.63/0.59	335–345, 363–371, 436–442
3	0.14/0.5	0.65/0.6	458–479
4	0.19/0.8		363–390

#### Docking and Virtual Screening

Our in-house library of generic drugs was selected to carry out virtual screening campaigns. The database was prepared with LigPrep module in order to generate energy-minimized 3D geometries by using OPLS 3 force field, plausible tautomers, stereoisomers, and protonation/ionization states at pH 7 ± 2 trough Epik.^52^

For virtual screening, Glide,^53,54^ implemented in Maestro, was selected as the first engine for docking. Prior to the docking step, four different receptor grids were generated, centered in sites 1, 2, 3 (refined structure from PDB: 6M0J), and 4 (refined structure from PDB: 6ZB5) previously identified on the RBD. Afterward, the virtual screening workflow implemented in maestro was chosen with two consequent different filters. The first stage of the virtual screening protocol encompassed the HTVS mode, in which up to 10% of best-scored molecules were selected for the next virtual screening stage. In the second step, SP (standard precision mode), the 10% of best poses, 29 different candidates were retained and selected for a thorough visual inspection, while the rest of the docking parameters were set as default.

As a second virtual screening tool, FlexX^55^ engine through the SeeSAR app interface (SeeSAR version 10.0; BioSolveIT GmbH, Sankt Augustin, Germany, 2020, https://www.biosolveit.de/SeeSAR) was selected to perform a consensus docking approach. For each molecule in the prepared library, poses were generated in the four different sites and then, predicted binding energy HyDE^56^ was calculated to rank all of the poses thus obtained. We selected the same number of top solutions as for Glide for further visual inspection except for site 4, in which we selected all candidates with an estimated affinity under the nanomolar range.

#### Molecular Dynamics Simulations

All-atom classical MD simulations of the RBD apo, RBD-glicosilated, and RBD-ligand bound complexes were performed using the AMBERff14sb,^57^ GLYCAM_06j-1,^58^ and GAFF2 ^59^ to describe proteins, glycans, and small molecules, respectively. All relevant disulfide bonds detected in the PDB as well as glycosidic linkages were specified as covalent connectivity. Antechamber program was used to calculate ligand atomic partial charges according to the AM1-BCC method.^60^ tLEaP program of AmberTools 16 was used to prepare the models for MD simulations.^61^ All systems were solvated in a truncated octahedral box of TIP3P water box leaving a minimum margin of 10 Å around the protein structure.^62^ Sodium and chloride ions were added until reaching a neutral net charge using the Li/Merz ion parameters. The MD preparation protocol was adapted from previous works, briefly several steps of energy minimization were performed: first to reorient all hydrogen and water molecules maintaining solute atoms restrained with a force constant of 5 kcal mol^–1^ Å^–2^ in 5000 steps of steepest descent, followed by side chain minimization for 4000 steps. Then, the whole system was minimized by performing 5000 steeps of steepest descent and 2500 of conjugated gradients. Afterward, the system was heated to 298 K for 100 ps applying positional restraint to Cα atoms of 2 kcal mol^–1^ Å^–2^. Then, the density was adjusted at 1 bat for 300 ps, maintaining the same positional restraints. Finally, 1 ns of NPT equilibration at 298 K and 1 bar was performed with the unrestrained systems. Molecular dynamics production was performed using a time step of 2.0 fs and applying SHAKE algorithm to all hydrogen bonds.^63^ Temperature control (298 K) was performed by means of Langevin dynamics, and pressure control was accomplished by coupling the system to a Berendsen barostat reference pressure of 1 atm. Periodic boundary conditions were applied and the particle mesh Ewald algorithm was used to compute long-range electrostatic interactions with a cutoff of 12 Å.^64^ Coordinates were stored in a trajectory file every 100 ps. The simulations were run in 50 ns blocks with coordinates of all atoms saved every 100 ps. Then, all files were concatenated and saved to a new trajectory every 5 snapshots to generate a whole solvated trajectory as well as a “dry” trajectory with all of the snapshots created during the production. At least two replicas of MD production were carried out for each system by means of the GPU-accelerated PMEMD engine implemented in AMBER.^100^ MD production simulations were run in the HPC Marconi100 using a Tesla V100-SXM2-16GB graphics card, yielding an average performance of 185 ns/day.

Trajectory analysis was performed with MDTraj,^65^ MDanalysis,^66^ and numpy^67^ Python libraries using the coordinates at frame 0 as a reference for alignment and analysis. Graphs were generated using Matplotlib Python module,^68^ while figures were generated via PyMOL (https://pymol.org), VMD,^69^ and UCSF ChimeraX.^70^

### Biophysical Studies

#### Site-Directed Mutagenesis and Protein Production

Plasmid pFastBac-Dual including the coding sequences for the SARS-CoV-2 RBD domain (residues Arg319–Phe541; Wuhan variant) in frame with an N-terminal gp67 signal peptide for secretion and a C-terminal 6× His tag for purification was used to produce RBD essentially as reported,^9^ by using the Bac-to-Bac Baculovirus Expression System (Invitrogen). Also on the plasmid described, the [S:G339D + S:S371L + S:S373P + S:S375F + S:S375F + S:K417N + S:N440K + S:G446S + S:S477N + S:T478K + S:E484A + S:Q493R + S:G496S + S:Q498R + S:N501Y + S:Y505H] substitutions were generated (Proteogenix) to produce the B.1.1.529.1­(BA.1) Omicron variant of the RBD. The correctness of the constructs, the presence of the desired mutations, and the absence of unwanted mutations were corroborated by Sanger sequencing.

The *N*-terminal peptidase domain of human ACE2 (residues Ser19–Asp615; hACE2) and the S:D614G variant of SARS-CoV-2 protein S ectodomain (residues 15–1213)^71,72^ were also produced in insect cells, using the plasmids and procedures described already. SARS-CoV-2 RBD (Wuhan or Omicron BA.1 variants) was purified as reported for SARS-CoV-2 protein S.^72^

In preparation for both thermofluor and thermophoresis assays, we generated the highly stable SpikeWuhan HexaPro,^73^ following the methods described elsewhere.^71,72^

#### Thermal Shift Assays

The thermal stability of SARS-CoV-2 RBD (Wuhan and Omicron BA.1 variants) and S protein (HexaPro) in the presence of selected compounds was evaluated using thermofluor assays,^74^ performed in sealed microwell plates as previously reported.^71,72^ Of all the compounds used in this study, only those dissolved in DMSO were used in this assay. These compounds were added at a final concentration of 500 μM to a 20 μL solution containing 0.7 μM RBD (0.017 mg/mL) or S (0.1 mg/mL) in 10 mM HEPES pH 7.2 and 0.15 M NaCl. After a 10 min incubation at 24 °C (room temperature), a 1:1000 dilution of SYPRO Orange (Invitrogen, Carlsbad, CA) was added. All assays (including those in the absence of compounds) were done in the presence of 5% DMSO. Additional controls measuring the intrinsic fluorescence of each compound in the absence of proteins were also carried out, and the signal obtained was subtracted from the total fluorescence signal of the protein in the presence of that compound. Experiments at final concentrations of fingolimod ranging from 100 to 500 μM were also done for RBD and spike proteins, as described above for 0.5 mM.

Fluorescence increase, reflecting protein unfolding, was monitored using a real-time PCR instrument (Applied Biosystems 7500 model, Thermo Fisher Scientific, Alcobendas, Madrid, Spain) by exciting SYPRO Orange at 488 nm and measuring emission at 610 nm as the temperature increased at a constant rate of 1 °C per minute. Each assay consisted of three replicate wells for each data point and was repeated at least twice on different days. GraphPad Prism 8 (GraphPad Software, San Diego, CA) was used for curve fitting, plot generation, and numerical analysis.

#### Microscale Thermophoresis (MST)

His-tagged SARS-CoV-2 RBD (Wuhan or Omicron) and S:HexaPro or S_Omicron_ proteins were labeled using His-Tag Labeling Kit RED-tris-NTA (NanoTemper Technologies) according to the manufacturer’s instructions. Briefly, and following our previously reported procedure,^71^ equal volumes of 200 nM target protein and 100 nM of dye solution were mixed and incubated for 30 min at room temperature followed by centrifugation at 10,000 rpm for 10 min. Both labeled RBD and Spike proteins were used at a final concentration of 50 nM. The assays were carried out in PBS pH 7.1 supplemented with 0.05% Tween-20. For the measurement of the binding affinity, a 16-point 2-fold dilution series (ranging from 5 mM to 0.15 μM) of the compound at 5% final DMSO concentration in the assay buffer was mixed with labeled proteins (1:1). Similarly, for the competition assays, each of the compounds was incubated at a final concentration of 500 μM with a mixture containing the target proteins and hACE2 protein at concentrations ranging from 5 μM to 0.15 nM in the same buffer. The mixture was incubated at room temperature before filling it in the Monolith Capillaries (MO-K022 NanoTemper Technologies), and MST measurements were performed on a Monolith NT.115 (NanoTemper Technologies). The results were analyzed using M.O. Affinity Analysis software (NanoTemper Technologies) as prescribed.^75^ All of the measurements were done in triplicates.

#### Negative-Stain Electron Microscopy

Spike complex (0.05 mg/mL S:D614G + 500 μM compound) was applied to a glow-discharged carbon-coated copper homemade grid for 30 s. The drop was removed using blotting paper, and the adsorbed proteins were negatively stained by applying three consecutive drops of 0.5% uranyl acetate and removing each drop with filter paper. Micrographs were collected on an Hitachi HT7800 microscope operated at 100 kV (Figure S7).

#### Biological Assays

VeroE6-TMPRSS2 (Catalog No. JCRB1819, Japanese Collection of Research Bioresources), VeroE6 (kindly provided by Dr. Luis Enjuanes, CNB–CSIC, Spain), and A549-Ace2-TMPRSS2 (Catalog No. a549-hace2tpsa, Invivogen) were cultured in DMEM high glucose, with glutamine, 10% FBS, and 1% penicillin/streptomycin. Selection media containing 1 mg/mL G418 (Catalog No. A1720, Sigma) or puromycin (0.50 μg/mL) and hygromycin (300 μg/mL) were included for culturing VeroE6-TMPRSS2 and A549-Ace2-TMPRSS2, respectively. Resazurin (CAS 62758-13-8) was purchased from Sigma-Aldrich (Catalog R7107) and dissolved in PBS. Antiviral assays were performed as previously described.^71^ Briefly, the ability of the compounds to block viral entry into cells was assessed using a GFP-expressing vesicular stomatitis virus pseudotyped with the S protein of the Wuhan-Hu-1 SARS-CoV-2 strain, Delta, Omicron BA.1, and SARS-CoV-1, generated as previously described.^71^ Initial antiviral testing was performed by incubating cells with the indicated compound concentrations, followed by the addition of 500–1000 focus-forming units of pseudotyped VSV. Following 16 h, the GFP signal in each well was quantified using a live-cell microscope (Incucyte SX5, Sartorius) and standardized to the average fluorescence observed in mock-treated wells. Subsequently, cell viability was evaluated by a resazurin reduction assay (Alamar Blue), adding resazurin to each well at a final concentration of 44 μM, incubating for 2 h at 37 °C, and reading fluorescence on a Tecan Spark microplate reader with an excitation of 535 nm and emission of 595 nm. The R drc package (version 3.0-1) was used to calculate 50% effective concentration (EC50) via a three-parameter log–logistic regression model (LL.3 model). Evaluation of antiviral activity against SARS-CoV-2 variant encoding the D614G S mutation was performed at the Biosafety Level 3 (BSL-3) facility of the Fundación para Fomento de Investigación Sanitaria y Biomédica (FISABIO) in Valencia, Spain, as previously described.^71^

## Supplementary Material



## Notes

Summary of docking results; extended information on MD simulations; ZINC codes and drug name of top-ranked molecules from virtual screening; 2D chemical structure of top ranked drugs; RMSD plots of MD simulations; electron micrographs; and schematic representation of the polysaccharide chain attached to Asn343 (PDF)
