# Supplementary material for: Exploring SARS-CoV‑2 Spike RBD Pockets as Targets for Generic Drugs: A Combined Computational, Biophysical, and Biological Approach
Source: ACS Omega. 2025 Aug 25;10(35):40190–207. doi: 10.1021/acsomega.5c05175 (PMC12423841; doi:10.1021/acsomega.5c05175)
Supplement: Supplementary file 1 [file ao5c05175_si_001.pdf]

Exploring SARS-CoV-2 Spike RBD Pockets as Targets for Generic Drugs. A Combined Computational, Biophysical, and Biological approach

Javier García-Marín,[1]* Clara Francés-Gómez,[2] Alicia Forcada-Nadal,[3,4] Anmol Adhav,[3] Clara Marco-Marín,[3] Vicente Rubio,[3,4] Alberto Marina,[3,4] José-Luis Llácer,[3,4] Ron Geller,[2] Sonsoles Martín-Santamaría[1]*

[1]Centro de Investigaciones Biológicas Margarita Salas (CIB), CSIC, Madrid 28040, Spain

[2]Institute for Integrative Systems Biology (I2SysBio), UV-CSIC, Paterna 46980, Valencia, Spain

[3]Instituto de Biomedicina de Valencia (IBV), CSIC, Valencia 46010, Spain

[4] Group 739 at the IBV-CSIC of the Centro de Investigación Biomédica en Red en Enfermedades Raras of the Instituto de Salud Carlos III (CIBERER-ISCIII), 28029-Madrid, Spain

E-mail: javier.garciamarin@uah.es; smsantamaria@cib.csic.es

Table S1. *Summary of docking results obtained with Glide during virtual screening campaigns in the four different sites.*

**Glide SP**	**Site 1**	**Site 2**	**Site 3**	**Site 4**
**Hits Retrieved**	29	30	29	34
**Upper Docking Score** (kcal/mol)	-5.274	-6.443	-5.079	-9.168
**Bottom Docking Score** (kcal/mol)	-7.733	-8.17	-6.17	-10.393

Table S2. *Summary of docking results obtained with FlexX embebed in SeeSAR software during virtual screening campaigns in the four different sites.*

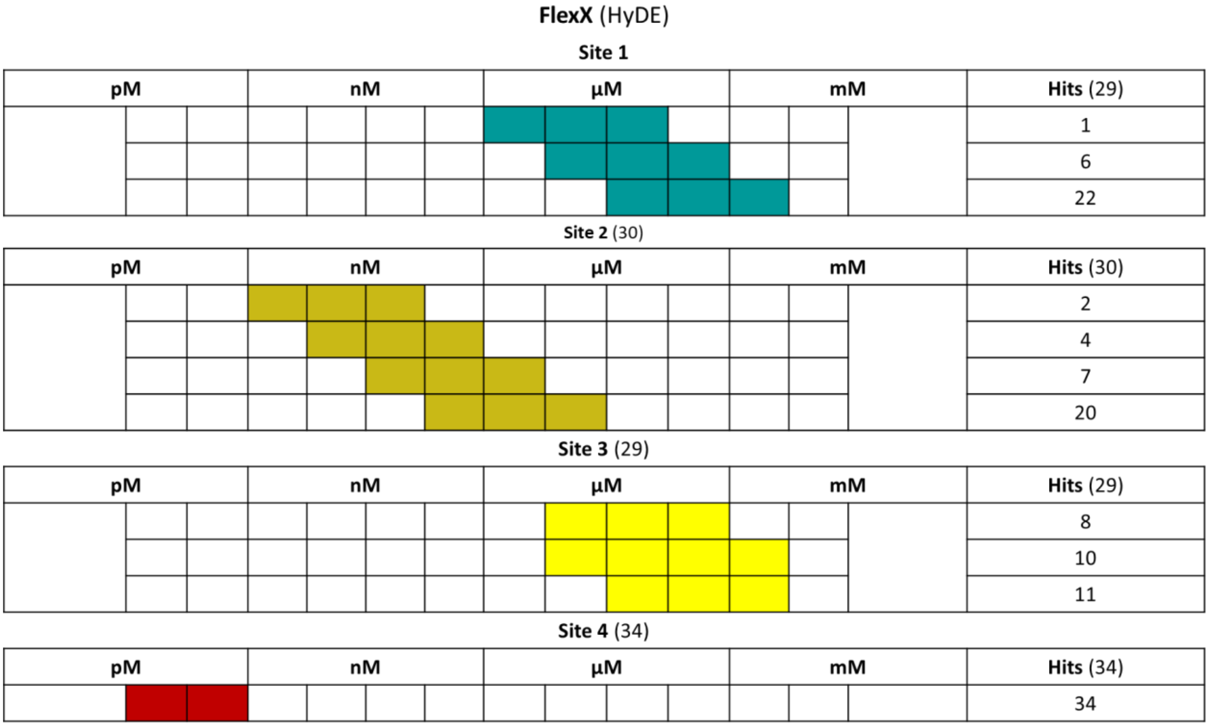

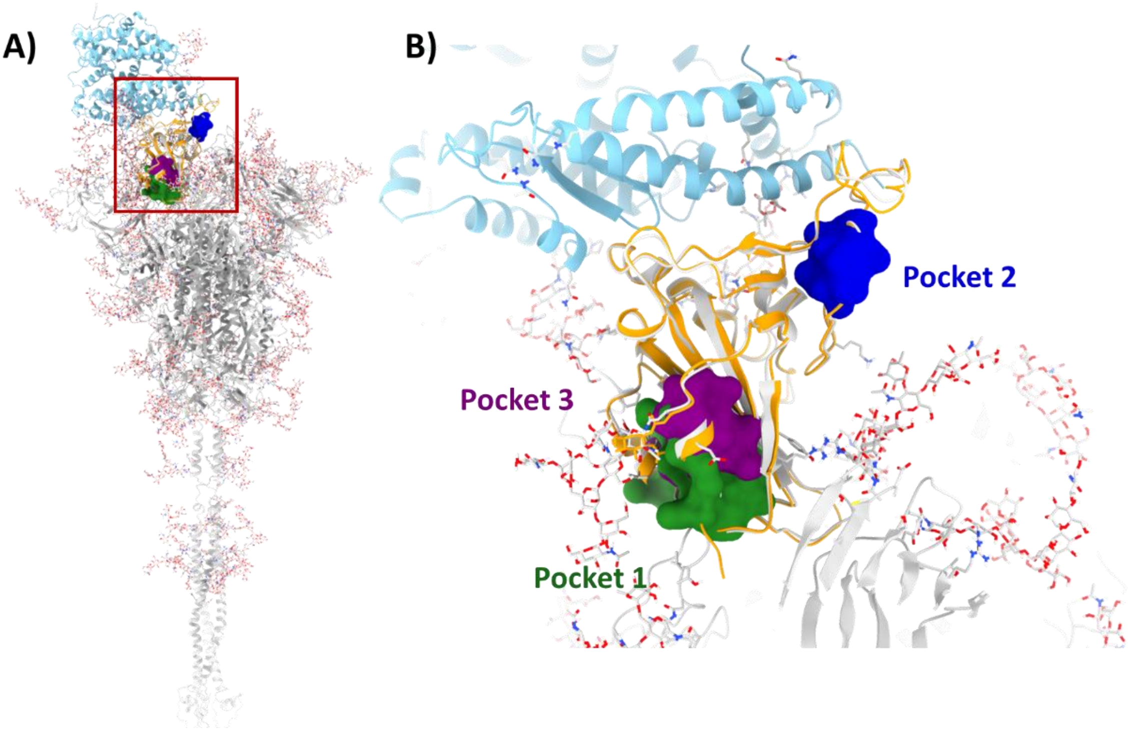

Figure S1. *A) Structure of the full-length glycosylated spike protein, with its Receptor Binding Domain (RBD) in an "up" conformation, complexed with ACE2 (retrieved from the CHARMMGUI repository: 6VSB_1_1_1). B) A detailed view of identified pockets, illustrating their potential accessibility for ligand binding.*

***Table S3**. Molecules selected for MD simulations from virtual screening. Residence time of a compound refers to how long (in ns) remained bound to the proposed binding site. For those molecules with residence time of 0 ns compound the compound escaped from the binding site during equilibration phase, prior production MD simulations.*

Drug	Comment	Residence time (ns) Replica 1	Residence time (ns) Replica 2
**Site 1**			
**Cefamandole**	Gram negative antibacterial	40	50
**Desferrioxamine B**	Iron chelant	450	300
**Fingolimod**	Treatment of multiple esclerosis	215	190
**Site 2**			
**Banzel**	Adjunct for Lennox-Gastaut syndrome	10	0
**Nabumetone**	Relief in rheumatoid arthritis and arthrosis	1000	1000
**Catechin**	-	1000	1000
**Hesperetin**	Cholesterol lowering flavanoid	1000	1000
**Calcifediol**	D deficiency or insufficiency, refractory rickets	1000	1000
**Ergocalciferol**	treatment of hypoparathyroidism, refractory rickets	1000	1000
**Fingolimod**	Multiple sclerosis treatment	1000	1000
**Betaxolol**	Adrenergic β1-antagonist	1000	-
**Salmeterol**	Adrenergic β2-agonist	1000	20
**Site 3**			
**Iohexol**	Contrast agent	700	50
**Glafenine**	Nonsteroidal anti-inflammatory	0	0
**Methoxamine**	Adrenergic α1-angonist	900	20
**Site 4**			
**Famprofazone**	Nonsteroidal anti-inflammatory	1000	1000
**Oxyphenonium**	Muscarinic acetylcholine receptor M1 antagonist	1000	1000
**Antrafenine**	Nonsteroidal anti-inflammatory	1000	1000
**Sertindole**	Antipsychotic for Schyzofrenia treatment	1000	1000
**Trazodone**	Treatment of major depressive disorder	1000	1000
**Toremifene**	Estrogen receptor alpha antagonist for breast cancer	1000	1000
**Fulvestrant**	Estrogen receptor alpha antagonist for breast cancer	1000	1000
**Flupentixol**	Antipsychotic for Schyzofrenia treatment	1000	1000
**Cholecalciferol (vitamin D3)**	D deficiency or insufficiency, refractory rickets	1000	1000

Table S4. *ZINC code and drug name of top ranked molecules from virtual screening in site 1.*

Glide		FlexX	
**ZINC CODE**	**Name**	**ZINC CODE**	**Name**
ZINC000008215403	NADH	ZINC1542002	Fingolimod
ZINC000005225520	Lactitol	ZINC643046	Persantine
ZINC000003977803	-	ZINC3872605	Fluvoxamine
ZINC000008577218	Folic acid	ZINC3830943	-
ZINC000238730526	-	ZINC1909090	Metvix
ZINC000003830635	Desferroxamine B	ZINC14007	Altol
ZINC000238730527	-	ZINC3911	Dobutamine
ZINC000072206342	-	ZINC1366	-
ZINC000003801919	Alendronate	ZINC1530759	Rythmol
ZINC000003776970	Cefamandole	ZINC20240	Propranolol
ZINC000004228235	Tetrahydrofolate	ZINC12358661	Endo-Atropine
ZINC000003861768	-	ZINC3944422	Norvir
ZINC000001530775	Pentamidine	ZINC1851149	Abilify
ZINC000003803652	Zoledronate	ZINC2149829	Clofoctol
ZINC000004534090	-	ZINC56645	Pindolol
ZINC000000012342	Ndga	ZINC3812918	Bepridil
ZINC000006627681	Trimethoprim	ZINC169342133	-
ZINC000001587572	Apga	ZINC22446685	Thioproperazine
ZINC000003830449	Cefoxitin	ZINC1530579	Coreq
ZINC000003803652	Zoledronate	ZINC18087	Almotriptan
ZINC000003977786	-	ZINC106	-
ZINC000004228237	Tetra-H-folate	ZINC6411960	-
ZINC000000002101	AVC	ZINC1530717	Methoprolol
ZINC000003860156	AMP	ZINC1529178	-
ZINC000003830569	Clavulanate	ZINC3819138	Dapaglifozin
ZINC000018043251	Theophylline	ZINC3820029	Trajenta
ZINC000008614810	Zoxazolamine	ZINC2031038	Fulsuramide
ZINC000003801919	Alendronate	ZINC3782550	Kerastick
ZINC000003801919	Alendronate	ZINC6411960	-

Table S5. *ZINC code and drug name of top ranked molecules from virtual screening in site 2*.

Glide		FlexX	
**ZINC CODE**	**Name**	**ZINC CODE**	**Name**
ZINC000150338697	Ocphyl	ZINC8214629	N-9
ZINC000150338696	Octeotride	ZINC3785268	Salmeterol
ZINC000004215219	Cefalonium	ZINC1530567	Betaxolol
ZINC000004215219	Cefalonium	ZINC3813024	Pipothiazine
ZINC000002008866	Zyvox	ZINC1530569	Bisoprolol
ZINC000001997127	Imid3	ZINC4538773	Aldmidrol
ZINC000000643114	Indapamide	ZINC2005163	Methoprene
ZINC000003830432	Cefoperazone	ZINC1530567	Betaxolol
ZINC000000968328	Rosiglitazone	ZINC00039091	Hesperetin
ZINC000000968328	Rosiglitazone	ZINC3646134	-
ZINC000169621234	Signifor	ZINC128	Carteolol
ZINC000000007782	Banzel	ZINC3830953	-
ZINC000003830449	Cefoxitin	ZINC538483	Trazodone
ZINC000008215434	FAD	ZINC11617039	Pazopanib
ZINC000003830428	Cefocinid	ZINC3785276	Epa-E
ZINC000000020221	Nabumetone	ZINC12484919	Calcidediol
ZINC000027990463	Iomitapide	ZINC1542002	Fingolimod
ZINC000150338696	Octeotride	ZINC19369227	Camylofin
ZINC000150338697	Ocphyl	ZINC2040210	Captodiame
ZINC000007997966	-	ZINC3972949	Ioflupane
ZINC000000119983	Catechin	ZINC407	Isoxsuprine
ZINC000000002159	Idt	ZINC3831417	Retinol
ZINC000000968326	Pioglitazone	ZINC14551	Ifenprodil
ZINC000000039092	Yso2	ZINC407	Isoxsuprine
ZINC000000011012	Ketorolac	ZINC250	Eperisone
ZINC000003830394	Cefamandole	ZINC407	Isoxsuprine
ZINC000003830434	Ceforanide	ZINC654	Pranoprofen
ZINC000003875368	Nitrofurantoin	ZINC1898	Penbutolol
ZINC000000001590	Hydrazide	ZINC3874604	Pantethine

Table S6. *ZINC code and drug name of top ranked molecules from virtual screening in site 3*.

Glide		FlexX	
**ZINC CODE**	**Name**	**ZINC CODE**	**Name**
ZINC000000057407	Methoxamine	ZINC607910	Glafenine
ZINC000150339323	Iohexol	ZINC607939	Isradipin
ZINC000003830990	Keppra	ZINC4214700	Paliperidone
ZINC000003830727	-	ZINC4214700	Paliperidone
ZINC000000895330	5-Hydroxytryptophan	ZINC13118910	-
ZINC000000001695	Metaraminol	ZINC113404	Teldrin
ZINC000003870129	Oxiglutatione	ZINC2015039	Benfluorex
ZINC000000057624	Adrenor	ZINC346	Cuvposa
ZINC000000113355	Phenylephrine	ZINC3871797	Bufexamac
ZINC000150339323	Iohexol	ZINC1678303	Isothipendyl
ZINC000000000558	Octopamine	ZINC4217587	Tolonium
ZINC000003798074	Acadesine	ZINC7673	Vimpat
ZINC000003803652	Zoledronate	ZINC608179	Anileridine
ZINC000060183170	Paramomycin	ZINC22010375	Phendimetrazine
ZINC000003803652	Zoledronate	ZINC666	Pronetalol
ZINC000060183170	Paramomycin	ZINC257	Esmolol
ZINC000038174297	-	ZINC53045054	Phenoxybenzamine
ZINC000003830944	Iohexol	ZINC13454202	Anisotropine
ZINC000003798064	Cladribine	ZINC22010375	Phendimetrazine
ZINC000096006041	Coenzyme A	ZINC22010375	Phendimetrazine
ZINC000000051581	Procodazole	ZINC1481789	Dimethindene
ZINC000000895457	Oxyproline	ZINC388662	-
ZINC000004340269	Amiloride	ZINC3785276	Epa-E
ZINC000000158585	Piconol	ZINC53045054	Phenoxybenzamine
ZINC000003830569	Clavulanate	ZINC81	-
ZINC000000895302	Mbn	ZINC81	-
ZINC000000001691	Metacetamol	ZINC18847040	Quinacrine
ZINC000038212689	Carac	ZINC215	Diethylpropion
ZINC000000056549	Hexestrol	ZINC19418959	Trifluoperazine

Table S7. *ZINC code and drug name of top ranked molecules from virtual screening in site 4*.

Glide		FlexX	
ZINC CODE	Name	ZINC CODE	Name
ZINC000012503187	Conivaptan	ZINC12404516	Toremifene
ZINC000000538337	Sertindole	ZINC3926298	Fulvestrant
ZINC000000538337	Sertindole	ZINC607861	Famprofazone
ZINC000072481725	Flupenthixol	ZINC1530866	Halofantrine
ZINC000012503187	Conivaptan	ZINC1530866	Halofantrine
ZINC000019418959	Trifluoperazine	ZINC3926298	Fulvestrant
ZINC000003995807	Fulvestrant	ZINC1530689	Tamoxifen
ZINC000004215219	-	ZINC3775140	Cisapride
ZINC000000538483	Trazodone	ZINC1529439	Alpha-Tochopherol
ZINC000019632628	Seroquel	ZINC3793063	-
ZINC000019632628	Seroquel	ZINC3926298	Fulvestrant
ZINC000000537755	Fluspiriline	ZINC1530695	Thioridazine
ZINC000000537755	Fluspiriline	ZINC3926298	Fulvestrant
ZINC000053073961	Antrafenine	ZINC1530973	Butoconazole
ZINC000053073961	Antrafenine	ZINC3926298	Fulvestrant
ZINC000100037132	Flupenthixol	ZINC1530167	Vitamin D3 (Cholecalciferol)
ZINC000000607861	Famprofazon	ZINC608024	Phenothrin
ZINC000000601274	Astemizole	ZINC3812888	Verapamil
ZINC000001542113	Vilazodone	ZINC1530973	Butoconazole
ZINC000001542113	Vilazodone	ZINC3831581	Trimethaphan
ZINC000100037135	Flupenthixol	ZINC3831581	Trimethaphan
ZINC000000538065	Nefazodone	ZINC608024	Phenothrin
ZINC000000601274	Astemizole	ZINC3831581	Trimethaphan
ZINC000019418959	Trifluoperazine	ZINC3831581	Trimethaphan
ZINC000004175630	Orap	ZINC53073961	Antrafenine
ZINC000004175630	Orap	ZINC1550766	Osphena
ZINC000019203912	Fluphenazine	ZINC8214402	Ibutilide
ZINC000019203912	Fluphenazine	ZINC968275	Trimipramine
ZINC000003831042	Mesoridazine	ZINC1481844	Fendiline
ZINC000003831042	Mesoridazine	ZINC1812122	Oxyphenonium
ZINC000001493878	Sorafenib	ZINC4212953	Dihydrotachysterol
ZINC000000537822	Haloperidol	ZINC968275	Trimipramine
ZINC000004213946	Nebivolol	ZINC808	Trimeprazine
		ZINC72481725	Flupenthixol
		ZINC1530695	Thioridazine
		ZINC3812975	Metergoline
		ZINC607861	Famprofazone
		ZINC4016817	-

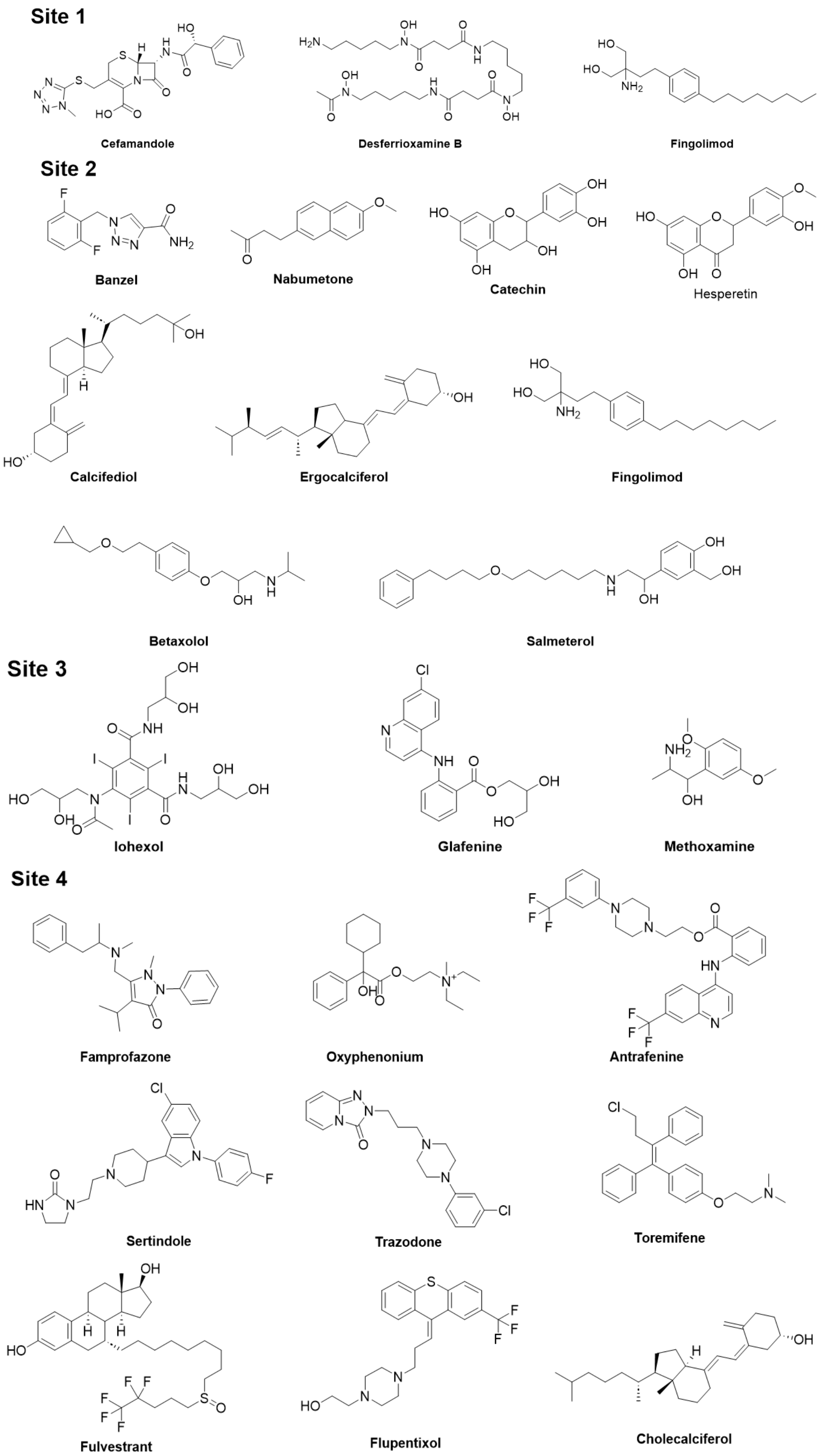

Figure S2. *2D Chemical structures of top docking drugs selected for MD simulations of their corresponding complexes with the RBD of the S protein.*

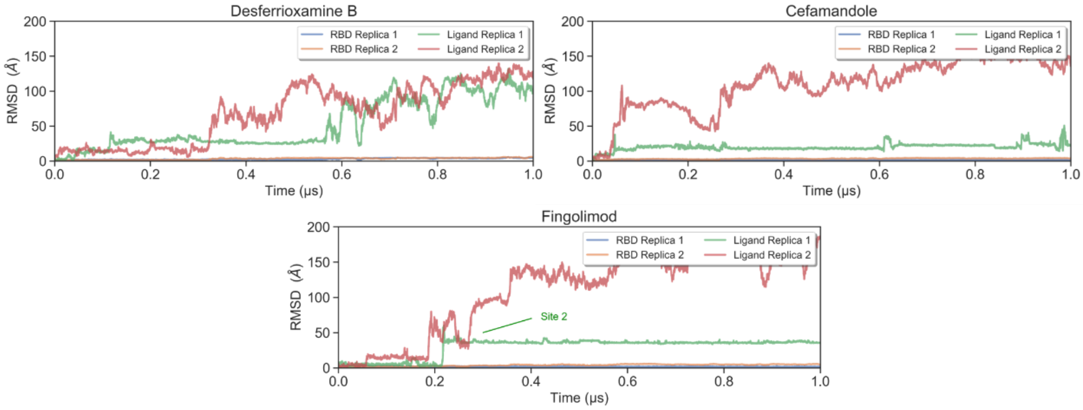

Figure S3. *RMSD plots of RBD Cα and ligand heavy atoms at site 1, for two MD simulation replicas. Independent simulations 1 and 2 are shown in blue and orange for the RBD and green and ref for ligand respectively.*

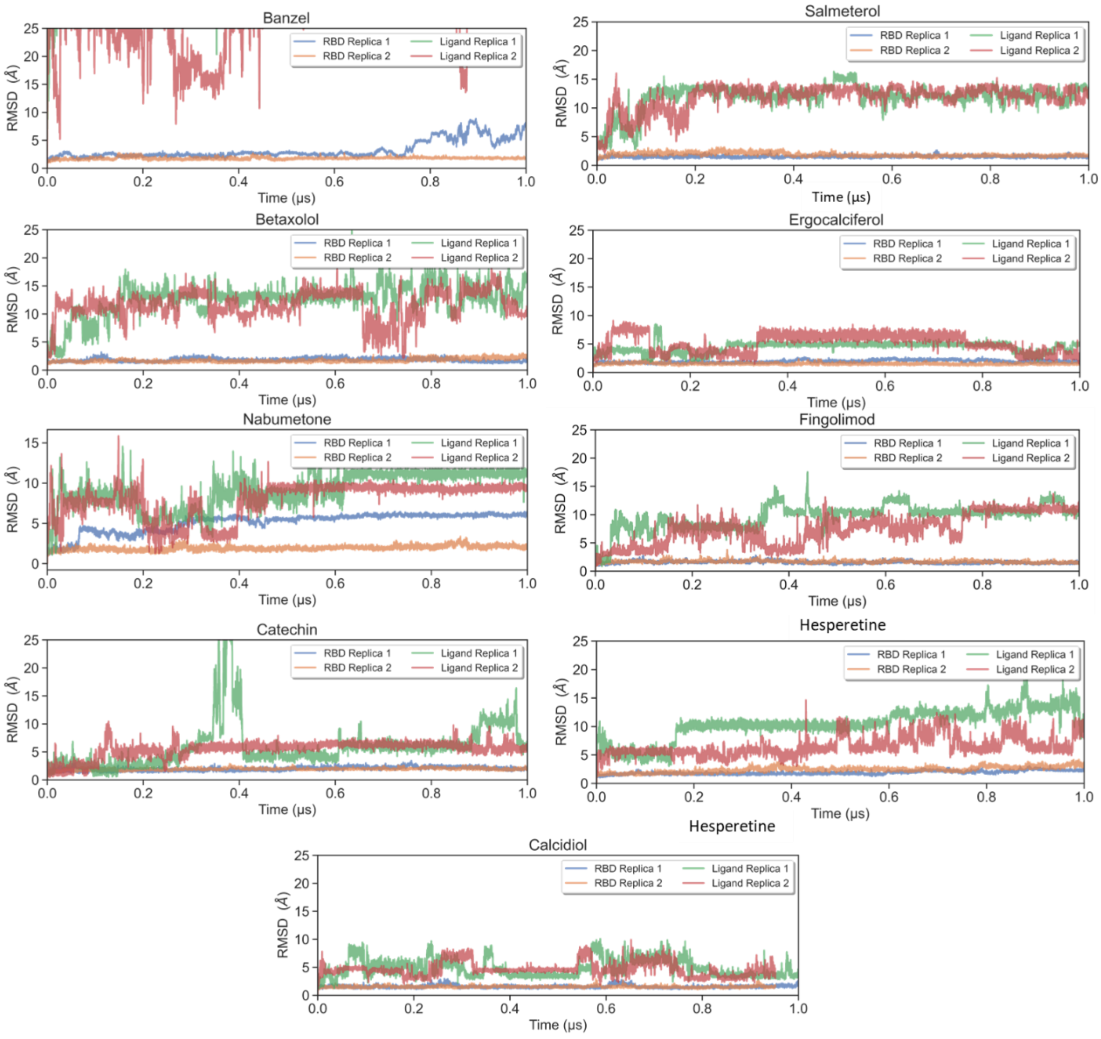

Figure S4. *RMSD plots of RBD Cα and ligand heavy atoms at site 2, for two MD simulation replicas. Independent simulations 1 and 2 are shown in blue and orange for the RBD and green and ref for ligand respectively.*

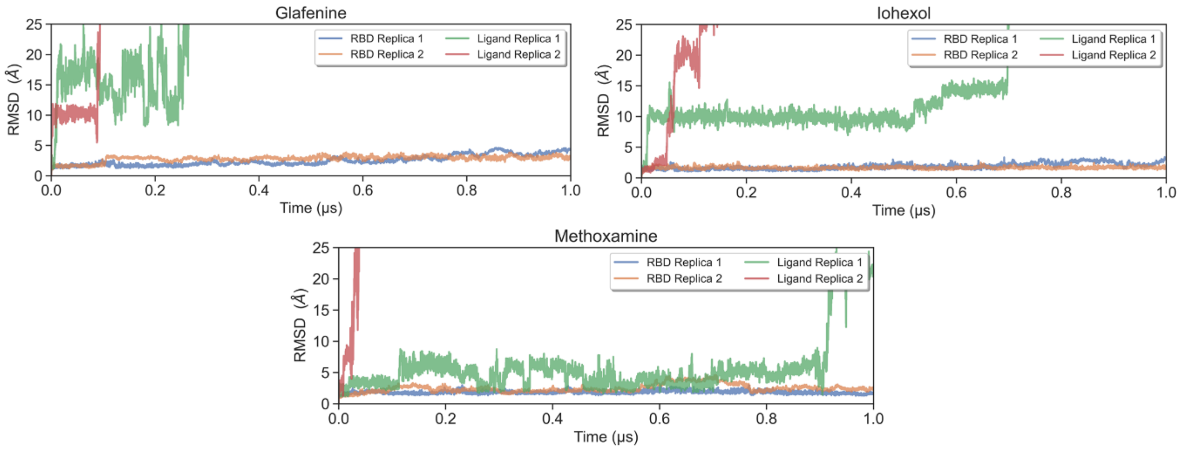

Figure S5. *RMSD plots of RBD Cα and ligand heavy atoms at site 3, for two MD simulation replicas. Independent simulations 1 and 2 are shown in blue and orange for the RBD and green and ref for ligand respectively.*

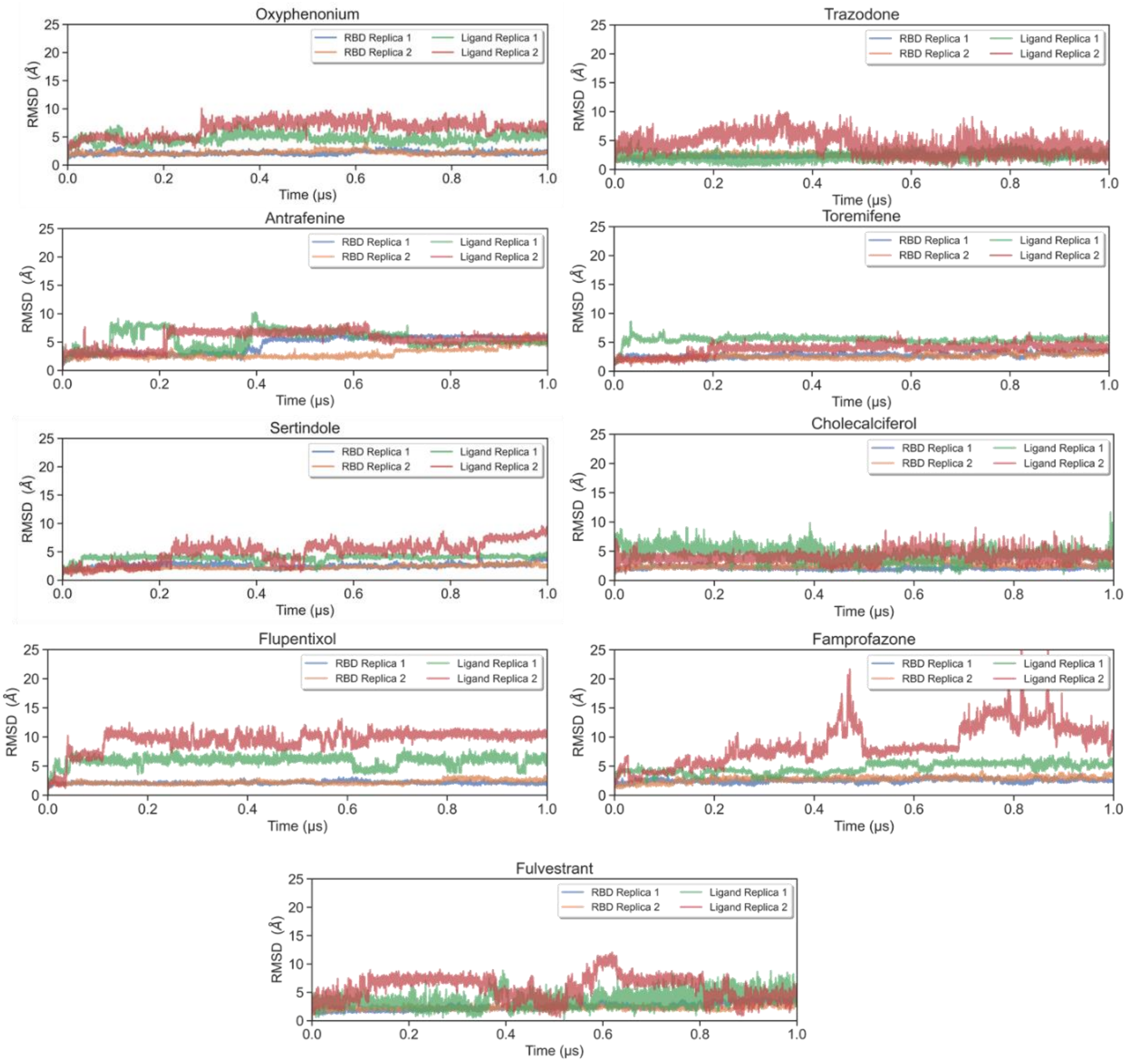

Figure S6. *RMSD plots of RBD Cα and ligand heavy atoms at site 4, for two MD simulation replicas. Independent simulations 1 and 2 are shown in blue and orange for the RBD and green and ref for ligand respectively.*

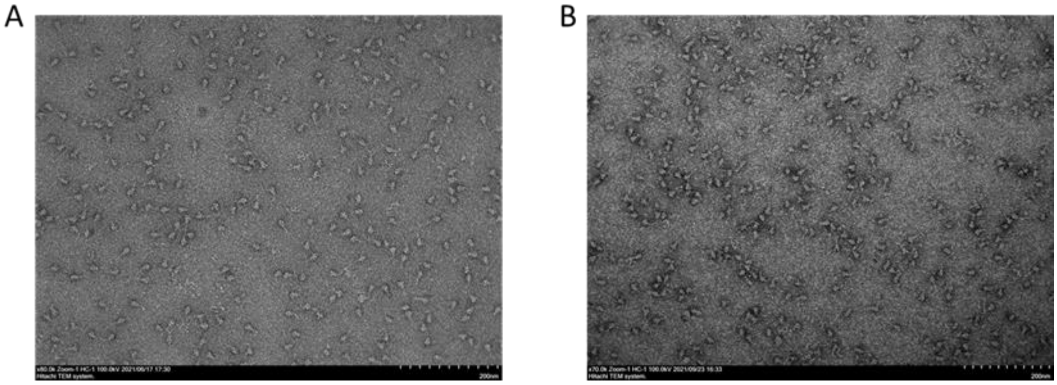

Figure S7. *Electron micrographs of negatively stained protein S (S:D614G) at 0.05mg/ml in the presence of 500µM cholecalciferol (A) or fingolimod (B). In both cases the S proteins were incubated at room temperature for 30 min with each compound previously to the preparation of the grids.*

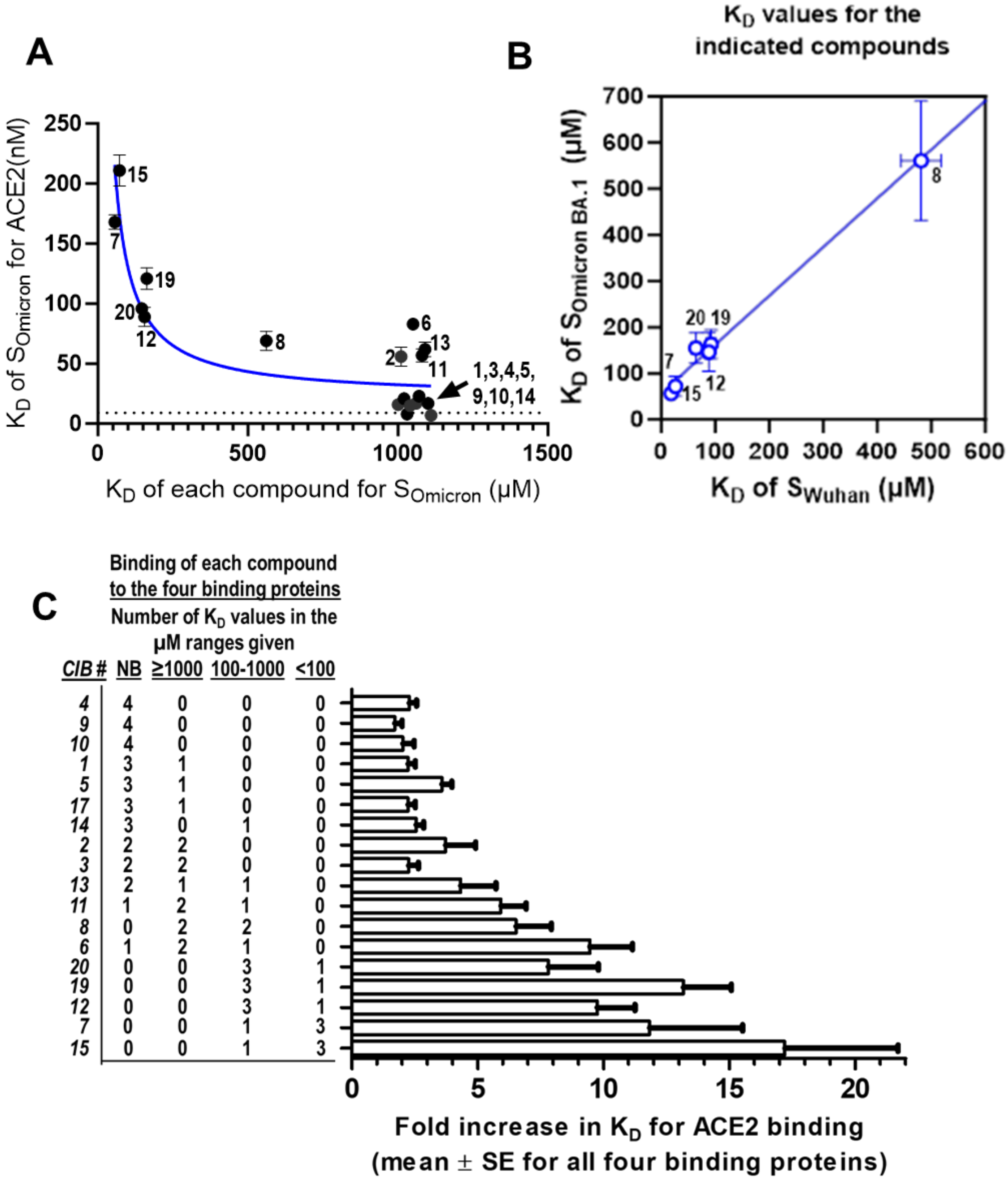

Figure S8. *Evidence that the compounds, by binding to the RBD and S proteins, interfere with the binding of these proteins to ACE2. (A) Plot of K_D apparent for the ACE2-$S_{Omicron}$ binding (mean ±SE) in the presence of 500 μM of the compounds that bind to the $S_{Omicron}$ with the K_D values shown in the X-axis. Compounds are identified numerically in the figure. Those with K_D values for $S_{Omicron}$ ≥1000 μM have been given slightly different values around 1000, for better visualization. The broken line correspds to upper limit of 95% CI in absence of compounds The curve fitted to the results follows the expected equation for competition: $K'_D{}^{ACE2}$(apparent)= $K_D^{ACE2}(1+500/K_D^{Compound})$, where x = $K_D^{Compound}$. (B) Linear correllation between K_D values of S_{Wuhan} and $S_{BA.1}$ for the different compounds (means ± SD; identified numerically). (C) Mean (±SE) n-fold increase in K_D values for ACE2 of the four proteins tested here, related to the strength of their binding of the compound employed, as schematized to the left of the plot.*

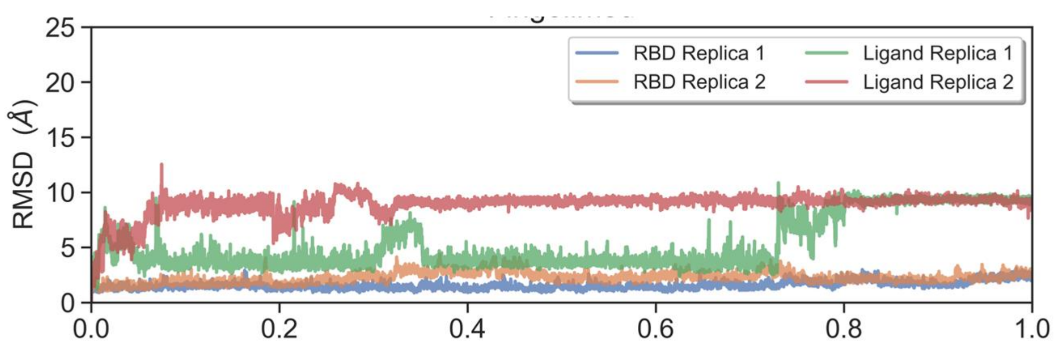

Figure S9. *RMSD plot of Omicron RBD Cα and fingolimod heavy atoms at site 2 during 1µs MD simulation replicas. B. Independent simulations 1 and 2 are shown in blue and orange for the RBD and green and ref for ligand respectively.*

bDGlcNAc(1→2)aDMan(1→6)[bDGlcNAc(1→2)aDMan(1→3)]bDMan(1→4)bDGlcNAc(1→4) [aLFuc(1→6)]bDGlcNAc(1→) -> ASN343

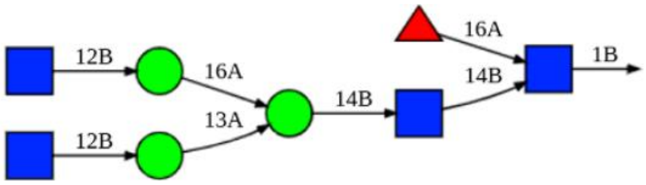

Figure S10. *Schematic representation of the polysaccharide chain attached to Asn343.*
